# IL-12 and GM-CSF engineered dendritic cells enhance the enrichment and selection of tumor-reactive T cells for cancer immunotherapy

**DOI:** 10.3389/fimmu.2025.1684842

**Published:** 2025-11-17

**Authors:** Zhen Liu, Haoyun Li, Zhaoya Gao, Ruoqi Cheng, Jiahua Lu, Xuan Che, Jiebin Dong, Zilong Wang, Zejia Cui, Jin Gu, Yun Bai, Cheng Li, Yinan Liu, Chengyan Wang, Hongkui Deng

**Affiliations:** 1 Department of Cell Biology and Stem Cell Research Center, School of Basic Medical Sciences, Peking University Health Science Center, Peking University, Beijing, China; 2 The Second Affiliated Hospital of Chongqing Medical University, Chongqing, China; 3 Department of Gastrointestinal Surgery, Peking University Shougang Hospital, Beijing, China; 4 School of Life Sciences, Center for Bioinformatics, Center for Statistical Science, Peking University, Beijing, China; 5 School of Life Sciences, Peking University, Beijing, China; 6 Department of Cell Biology and Stem Cell Research Center, School of Basic Medical Sciences, State Key Laboratory of Natural and Biomimetic Drugs, Peking University Health Science Center, Peking University, Beijing, China; 7 Changping Laboratory, MOE Key Laboratory of Cell Proliferation and Differentiation, College of Life Sciences, Peking-Tsinghua Center for Life Sciences, Peking University, Beijing, China

**Keywords:** reactive T cells, DC, enrichment and selection, IL-12, GM-CSF

## Abstract

The use of tumor-reactive T cells in targeted tumor elimination holds significant potential for cancer immunotherapy, such as Tumor-Infiltrating Lymphocyte (TIL) therapy and TCR-T adoptive immunotherapy. Critical aspects of the effective clinical application of these immunotherapies include the enrichment and selection of tumor antigens and their corresponding reactive T cells. However, current *in vitro* methods for expanding and screening tumor antigen-reactive T cells remain inefficient. One reason for this inefficiency is the dysfunctional state of tumor-reactive T cells, which limits their expansion and activation. To address this challenge, we developed an optimized dendritic cell-based culture system, in which dendritic cells simultaneously express interleukin-12 and granulocyte-macrophage colony-stimulating factor (12GM-DCs), to enhance the expansion of tumor-reactive T cells. We found that 12GM-DCs can enrich reactive T cells targeting various tumor antigens, including virus-associated tumor antigens, tumor-associated antigens, mutant tumor neoantigens, and patient-specific tumor neoantigens. Moreover, 12GM-DCs increased the proportion of antigen-specific T cells, enhanced the activation of those T cells, and promoted the maintenance of a memory phenotype. The cytotoxicity of these antigen-reactive T cells was increased after co-culture with 12GM-DCs, likely due to the increased secretion of interferon-γ and granzyme B. Importantly, these functions and phenotypic advantages of tumor antigen-reactive T cells derived from the 12GM-DC culture system could be effectively maintained and the antitumor activity was also enhanced in tumor-burden mice. Our 12GM-DC coculture system effectively enriches antigen-specific T cells and has the potential to advance the clinical application of cancer immunotherapy by targeting tumor antigens and their reactive T cells.

## 1 Introduction

For patients with advanced solid tumors, high tumor heterogeneity constitutes a substantial challenge to both diagnosis and treatment (1). Adoptive cell transfer therapy, which relies on the identification of tumor-reactive antigens and expansion of tumor-reactive T cells, has emerged as a promising approach for efforts to develop cancer immunotherapies aimed at curing solid tumors (2). After amplification and enrichment, patient-specific antigen-reactive T cells have been transplanted, leading to optimal tumor control. In a clinical trial, three patients with metastatic breast cancer exhibited objective tumor regression after adoptive therapy using neoantigen-selected reactive T cells, illustrating the effectiveness of this tumor treatment approach (3). In two additional trials (4, 5), patients infused with neoantigen-reactive TCR-T cells experienced durable partial responses.

Although the transfers of expanded tumor antigen-specific T cells are effective approaches for solid tumor treatment, several key challenges remain. The identification and validation of tumor antigens depend on *in vitro* functional assays to determine the reactivity of T cells that recognize these antigens (6–8). The screening and identification of tumor antigens and their corresponding specific T-Cell Receptors (TCRs) are difficult and inefficient processes. A key factor contributing to this difficulty and inefficiency is that tumor-reactive T cells cultured in conventional systems often enter a dysfunctional state, losing their effector function, exhibiting reduced proliferative capacity, displaying increased expression of inhibitory receptors, and becoming susceptible to apoptosis (9). There is a critical need to develop an optimized culture system for tumor-reactive T cells to address these issues, which would facilitate the implementation of antigen screening for solid tumor therapies.

In recent years, cytokine-based anti-cancer therapy has emerged as a promising approach because cytokines have multiple effects on immune cells within tumors (10). Cytokines mediate intercellular communication and play important roles in promoting immunity or inhibiting cell differentiation and proliferation. Among the known cytokines, Interleukin (IL)-12 has been identified as a robust candidate to enhance T cell immunity. IL-12 stimulates T cell proliferation, differentiation, and antigen responsiveness (11). Additionally, IL-12 activates T cells by upregulating the expression of adhesion molecules and stimulating the transcription of perforin and granzymes. IL-12 also induces Interferon (IFN)-γ production by T cells, a crucial factor in anti-tumor responses (12). Another important cytokine is Granulocyte-Macrophage Colony-Stimulating Factor (GM-CSF), which has been shown to promote anti-tumor immune responses by activating monocytes and macrophages, while enhancing Dendritic Cell (DC) differentiation (13). GM-CSF induces the upregulation of Human Leukocyte Antigen-DR (HLA-DR) and CD86; it also increases the secretion of tumor necrosis factor-α and IL-1β. Furthermore, GM-CSF enhances the initiation and activation of T cells in the spleen and lymph nodes by increasing IFN-γ secretion (14). GM-CSF-deficient mice exhibit impaired T cell proliferation and IFN-γ production, and they fail to generate antigen-specific CD8^+^ T cells (15).

Considering the central roles of IL-12 and GM-CSF in regulating T cell immunity, we aimed to generate DCs co-expressing IL-12 and GM-CSF (12GM-DCs) that would enhance the culture of activated and enriched tumor-reactive T cells. We produced 12GM-DCs through adenoviral transduction, followed by co-culture of the 12GM-DCs with T cells *in vitro*. The functions of T cells in this co-culture system were evaluated. We systematically assessed tumor virus-associated antigen, Tumor-Associated Antigen (TAA), tumor-mutated neoantigen, and patient-specific tumor neoantigen-reactive T cells in a modified 12GM-DC co-culture system. We observed enhanced activation, enrichment, and memory phenotype in tumor-reactive T cells. This optimized culture system offers a platform for enriching and screening tumor antigen-reactive T cells, thereby advancing the development of novel cancer immunotherapies for solid tumors.

## 2 Methods

### 2.1 Cell culture

COS7 cells, derived from African green monkey kidney fibroblasts and transformed with SV40 T virus gene, were cultured in Dulbecco’s Modified Eagle Medium (DMEM) containing 10% Fetal Bovine Serum (FBS; 900-108, Gemini, USA). HEK-293T and HEK-293A cells were cultured in DMEM with 10% FBS. All cells were maintained in an incubator at 37 °C with 5% CO_2_.

### 2.2 Construction of plasmids and production of lentivirus and adenovirus

The DNA sequences of GFP and 12GM were synthesized and subcloned into the pHBAd adenovirus. In pHBAd-12GM, the full genes of GM-CSF and IL-12 are included, with a 2A linker in between. Adenovirus is packaged and produced by transfecting HEK-293A cells, and the virus liquid is obtained by breaking the cells. The DNA sequences of 1G4 and TP53 TCR were synthesized, and subcloned into the pRRLSINcPPT lentiviral vector. Prepare the lentivirus supernatant using HEK-293T cells, as described previously (16).

### 2.3 Preparation of COS7 target cells

COS7 cells were co-infected with lentiviruses encoding HLA-A0201, CD80, CD83, CD86, and luciferase. After co-infection, the COS7 cells expressing these markers were sorted to serve as antigen-presenting cells. These COS7 antigen-presenting cells were then infected with either pRRL-EF1A-NY-ESO-1 or pRRL-EF1A-TP53 lentiviruses, and positive cells were sorted to obtain COS7 target cells.

### 2.4 Preparation of TCR-T cells

Healthy donor peripheral blood was obtained from the Red Cross Blood Center. TCR-T Cells were prepared as previously described (16). Peripheral Blood Mononuclear Cells (PBMCs) were isolated through gradient centrifugation using lymphocyte separation medium. The PBMCs were activated using anti-CD3/CD28 magnetic beads and cultured in X-VIVO medium (04-418Q, Lonza, CA, USA) supplemented with 10% FBS and 100 U/mL IL-2 (200-02, PeproTech, NJ, USA). Cells were maintained in an incubator at 37 °C with 5% CO_2_. After 48 hours of activation, an equal volume of lentiviral stock solution was added to infect the cells. The TCR-T cells were then continuously cultured and expanded *in vitro*; the medium was replaced every 2–3 days.

### 2.5 Culture of human-induced DCs

Induced DCs were generated from PBMCs. Briefly, adherent PBMCs were collected and re-cultured in X-VIVO medium supplemented with 10 ng/mL IL-4 and 1000 IU/mL GM-CSF (200-04, 300-03, PeproTech). The cells were incubated on the standard culture dish at 37 °C with 5% CO_2_. After 6–8 days, human-induced DCs were harvested by adherent digestion, and subsequently infected with adenovirus to generate 12GM-DCs and GFP-DCs.

### 2.6 Screening of patient-specific neoantigen-reactive T cells

Tumor and PBMC samples were collected from patients with colorectal cancer in our clinic. PBMCs were used for *in vitro* induction and culture of DCs, as well as T cell isolation. Concurrently, whole exome sequencing and RNA transcriptome sequencing were conducted to identify tumor mutations and HLA subtypes. Tumor biopsies and normal PBMC were subjected to DNA extraction, library construction, exome capture of approximately 20,000 coding genes and Next-generation sequencing by Macrogen (Rockville, MD), Personal Genome Diagnostics (PGDX, Baltimore, MD) or the Broad Institute (Cambridge, MA). An mRNA-sequencing library was also prepared from a tumor biopsy using Illumina TruSeq RNA library prep kit. The levels of transcripts encoding putative nonsynonymous variants were used to assess expression of candidate mutations identified using whole-exome data. Binding affinities between peptides generated from tumor mutations and each patient’s HLA molecules were predicted and scored using netMHCpan. From this analysis, 24–63 candidate peptides with the highest scores were selected for synthesis. These peptides [10.0 μg/mL; refer to published articles (17)] were co-cultured with DCs and T cells to enrich and expand tumor-reactive T cells. The enriched T cells were then restimulated with the neoantigen peptides. After 18–24 hours, activated T cells were detected using methods such as CD137 flow cytometry to identify tumor-reactive T cells. Additionally, single T cells with activated phenotypes were sorted by flow cytometry for TCR single-cell Reverse Transcription Polymerase Chain Reaction (RT-PCR) sequencing (17).

### 2.7 Flow cytometry detection of cell surface markers

Cells were collected, stained with fluorochrome-labeled antibodies, and incubated at 4 °C for 30 minutes. After incubation, the cells were washed by centrifugation and resuspended in phosphate-buffered saline, then analyzed by flow cytometry. Data were processed using CytExpert software. For flow cytometry analysis, we used the following antibodies purchased from BioLegend (CA, USA): anti-human CD137 (309810), anti-human OX40 (350012), anti-human CD28 (983404), anti-human CD27 (986906), anti-human CD80 (305219), anti-human CD83 (305305), anti-human CD86 (374205), anti-human CD45RO (983102), anti-human CD62L (980706), and anti-human CCR7 (353241).

### 2.8 Flow cytometry detection of TCR positive T cells

T cells were washed by centrifugation and resuspended in Phosphate-Buffered Saline (PBS). The proportion of mCherry-positive cells was then analyzed by flow cytometry with Cytoflex S (Beckman Coulter, Brea, CA). Data were processed using CytExpert software (Beckman Coulter, Brea, CA).

### 2.9 T cell cytotoxicity assay

Target cells overexpressing luciferase were seeded in an opaque 96-well plate (136101, Thermo, MA, USA). For tumor virus-associated antigen-reactive T cells, target cells were pulsed with peptides. For TAA-reactive and neoantigen-reactive T cells, target cells were infected by antigen-expressing lentivirus. After 12 hours, total T cells co-cultured with antigen-loaded 12GM-DCs or GFP-DCs were added to the plate at various ratios relative to target cells (1:1, 3:1, and 9:1) and co-cultured with the target cells. After 16–18 hours of co-culture, luciferin was added, and bioluminescence was measured within 10 minutes using a luminometer (LB960, Berthold, Germany).

### 2.10 Cytokine measurement

Cytokine levels in the culture supernatant or serum were measured using ELISA kits (Youda), in accordance with the manufacturer’s instructions. Enzyme-linked immunosorbent assay (ELISA) kits for hIL-12 (1111202), hGM-CSF (1117302), hIFN-γ (1110002), and human granzyme B (1118502) were obtained from Youda.

### 2.11 Detection of cell viability by cell counting kit-8 method

Place the cells into a 96-well plate and cultivate them under the predetermined culture conditions for the predetermined time. Add 10 μL of CCK-8 reagent to each well and return it to the 37°C, 5% CO_2_ cell incubator. After 2 to 4 hours, detect the OD value at 450 nm using an enzyme-linked immunosorbent assay reader.

### 2.12 Cell proliferation was measured by CFSE

Cells were harvested, stained with CFSE according to reagent instruction, incubated at 37°C for 10 min, and immediately added to PBS with 1%FBS. After centrifugation, the washed cells were cultured at 37°C for 48 h, and then the cells were collected for flow detection of FITC channel, and the data were analyzed by CytExpert software.

### 2.13 Cytokine release detected by ELISPOT

Monocyte-induced DCs and T cells were cultured in 96-well plates in the IFN-γ ELISPOT kit (Youda) and returned to the 37°C, 5% CO2 cell incubator. After 16–18 h, the cell culture suspension was removed and the number of IFN-γ spots was detected by ELISPOT kit.

### 2.14 *In vivo* anti-tumor experiments of co-culture enriched T cells in mice

NOD.Cg-Prkd^cscid^Il2rg^tm1^Vst (NPG) male mice aged 4–6 weeks were purchased from Beijing Vitalstar Biotechnology. The NPG mouse model was established by injecting 1×10^6^ SKOV3 cells overexpressing NY-ESO-1 under the armpit of one side of the mouse. When the tumor volume reached about 100 mm^3^, the mice were divided into groups according to tumor size. Each mouse received a tail vein infusion of 1×10^6^ coculture-enriched T cells or PBS. Every two days after T-cell infusion, mouse tumor size was measured, and mice were sacrificed by cervical dislocation method when the tumor volume approached 1000 mm^3^, and tumors were removed for photography. At the same time, on day 3 and day 7 after infusion, blood samples were collected from the inner canthus of the mice for flow cytometry to detect the maintenance of human T cells in the peripheral blood, and to detect the proportion, activation and memory phenotype of antigen-specific TCR-T cells.

### 2.15 Detection of peripheral blood T cells by flow cytometry

Around 200 μL peripheral blood from the inner canthus of the mice were collected and were centrifuged to remove the supernatant. Red blood cells were lysed by red blood cell lysate (R1010). Cells were collected and stained with fluorescein-labeled antibodies for 40 min at 4°C. Cells were washed twice with PBS and then analyzed by flow cytometry. The data were analyzed by CytExpert software.

### 2.16 RNA sequencing and data analysis

DCs were collected 48 hours after adenovirus infection, and RNA was extracted. Co-culture cells were collected after co-culture, and RNA was extracted. The RNA samples were sent to Beijing Novogene Technology Company for RNA sequencing. Subsequently, data analysis was performed as follows: First, FastQC was used for quality control of the raw sequencing data. Then, STAR was employed for read alignment and quantification. Following this, DESeq2 was applied for differential gene expression analysis. In parallel, Gene Ontology (GO) analysis was conducted for pathway enrichment assessment. The RNA transcriptome data was deposited in GSA public repository, and the accession number is HRA013815. Shared URL: https://ngdc.cncb.ac.cn/gsa-human/s/5h4JP28M.

### 2.17 HLA subtypes sequencing

Sequenced the HLA subtypes of the infused T cells by Beijing Capital Biotechnology Co., Ltd.

### 2.18 Statistical analyses

All experiments were performed at least three times. Data are presented as the mean ± standard deviation. Student’s t-test was used to assess significant differences between groups; p-values < 0.05 were considered statistically significant. “ns” indicates no significant difference; *p < 0.05; **p < 0.01; ***p < 0.001. Differences among more than two groups were determined by one-way ANOVA with a Tukey–Kramer multiple-comparison test.

### 2.19 Ethics approval and consent to participate

The clinical patient related experiments involved in this study were approved by the Ethics Committee of Peking University Shougang Hospital (IRBK-2017-034-01). The animal-related experiments involved in this study were approved by the Biomedical Ethics Committee of Peking University (LA2021541).

## 3 Results

### 3.1 Generation of DCs expressing IL-12 and GM-CSF

**Figure 1:**
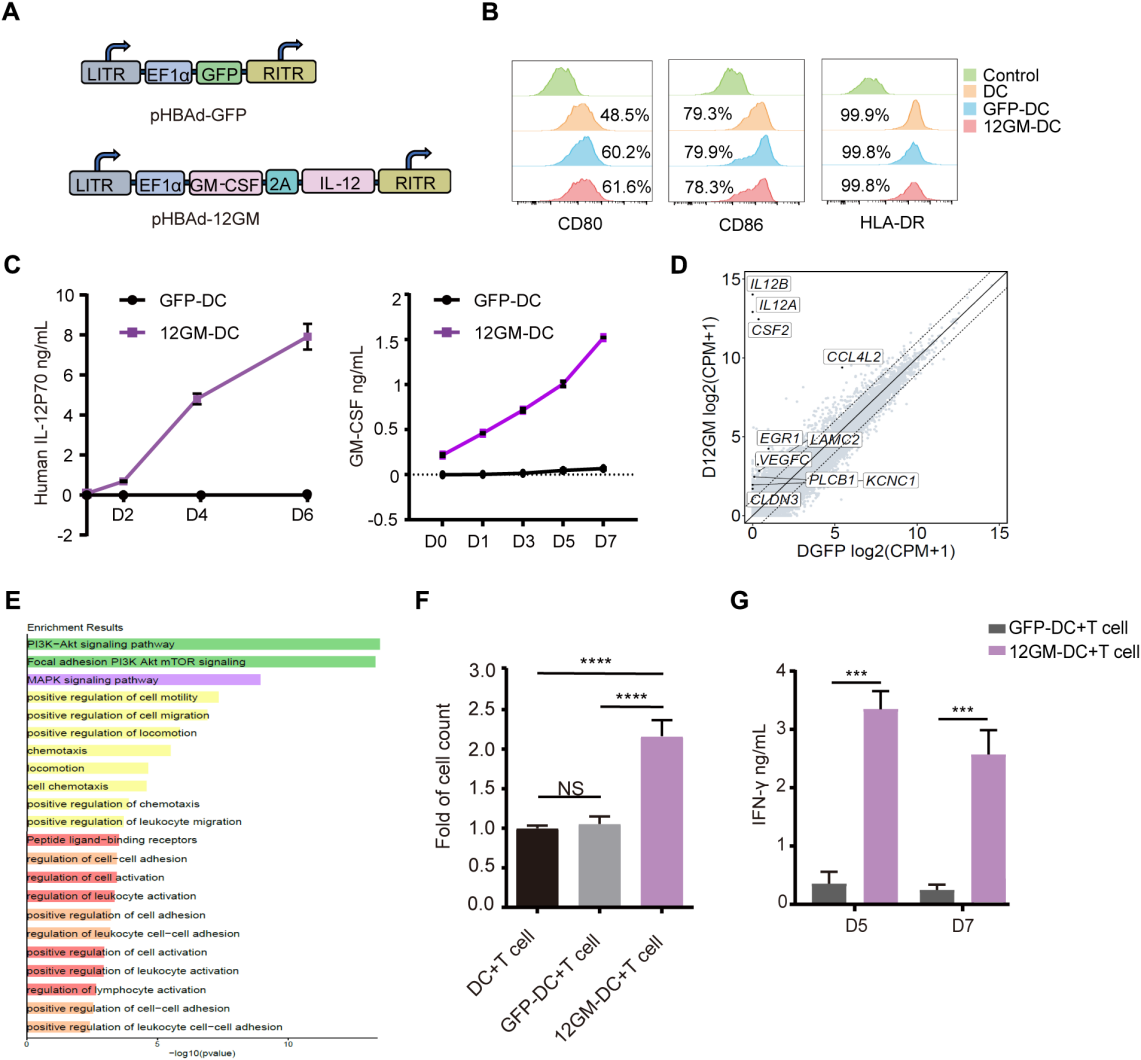
Construction of 12GM-DCs and identification of their functions. **(A)** Schematic diagrams of the structures of the 12GM adenovirus and GFP adenovirus. **(B)** The expression patterns of CD80, CD86, and HLA-DR on DCs infected with GFP and 12GM adenoviruses were detected by flow cytometry. **(C)** Levels of IL-12 and GM-CSF secretion from GFP-DCs and 12GM-DCs were measured by ELISA at 0, 24, 72, and 144 hours after adenovirus infection. After DCs, GFP-DCs, and 12GM-DCs had each been co-cultured with T cells for 1 week. **(D)** After 48 hours of adenovirus infection, the analysis of upregulated genes in the 12GM-DC transcriptome was conducted. **(E)** After 48 hours of adenovirus infection, the GO diagram for the upregulated pathways in the 12GM-DC transcriptome was analyzed. **(F)** The number of T cells was measured using the CCK8 assay, and **(G)** IFN-γ secretion from T cells was measured by ELISA. Data are presented as the means ± SEMs. ***P < 0.001, ****P < 0.0001. The experiment was repeated at least three times.

In this study, we generate DCs through inducing monocytes *in vitro* and modified them through adenoviral transduction. The adenovirus was constructed to carry sequences for IL-12 and GM-CSF, linked by P2A (Ad-12GM) (**Figure 1A**). Both Ad-GFP and Ad-12GM were used to infect the *in vitro*-induced DCs. Flow cytometry demonstrated the successful induction of DCs, and confirmed that all DCs expressed CD80, CD86, and HLA-DR (**Figure 1B**, **Supplementary Figure S1A**). ELISAs showed that GFP-DCs secreted minimal levels of IL-12 and GM-CSF, whereas 12GM-DCs secreted high concentrations of both cytokines. The concentrations of these cytokines gradually increased over time and remained elevated until day 6 (**Figure 1C**, **Supplementary Figure S1C**). Previous studies have shown that IL-12 stimulates T cell proliferation and activation; it also promotes the production of IFN-γ by T cells (18). Additionally, GM-CSF enhances T cell initiation and activation by increasing IFN-γ secretion (14). Meanwhile, transcriptome analysis revealed that genes and pathways related to cell activation, adhesion, and migration were upregulated in 12GM-DC (**Figures 1D, E**, and **Supplementary Figure S1E**). To evaluate the effect of 12GM-DCs on T cell proliferation, we co-cultured DCs, GFP-DCs, or 12GM-DCs with autologous T cells (**Supplementary Figure S1B**), then assessed cell proliferation on day 5 using the CCK8 assay. The results showed no significant change in the number of T cells after co-culture with GFP-DCs, whereas the number of T cells increased approximately two-fold after co-culture with 12GM-DCs (**Figure 1F**). At the same time, the proportion of T cell proliferation in 12GM-DC group was higher (**Supplementary Figure S1D**). Next, we performed ELISAs to assess the impact of 12GM-DCs on T cell cytokine secretion. The results indicated that co-culturing 12GM-DCs with T cells significantly enhanced the secretion of IFN-γ, reaching levels 10 times higher than those achieved by GFP-DCs (**Figure 1G**). And the T cells that were cultured with 12GM-DC showed a decrease in PD-1 expression compared to those that were co-cultured with GFP-DC. Consistent with previous findings (12, 14), we demonstrated that 12GM-DCs effectively promote T cell proliferation and IFN-γ secretion.

### 3.2 12GM-DCs optimize the enrichment of tumor virus-associated antigen-reactive T cells

**Figure 2:**
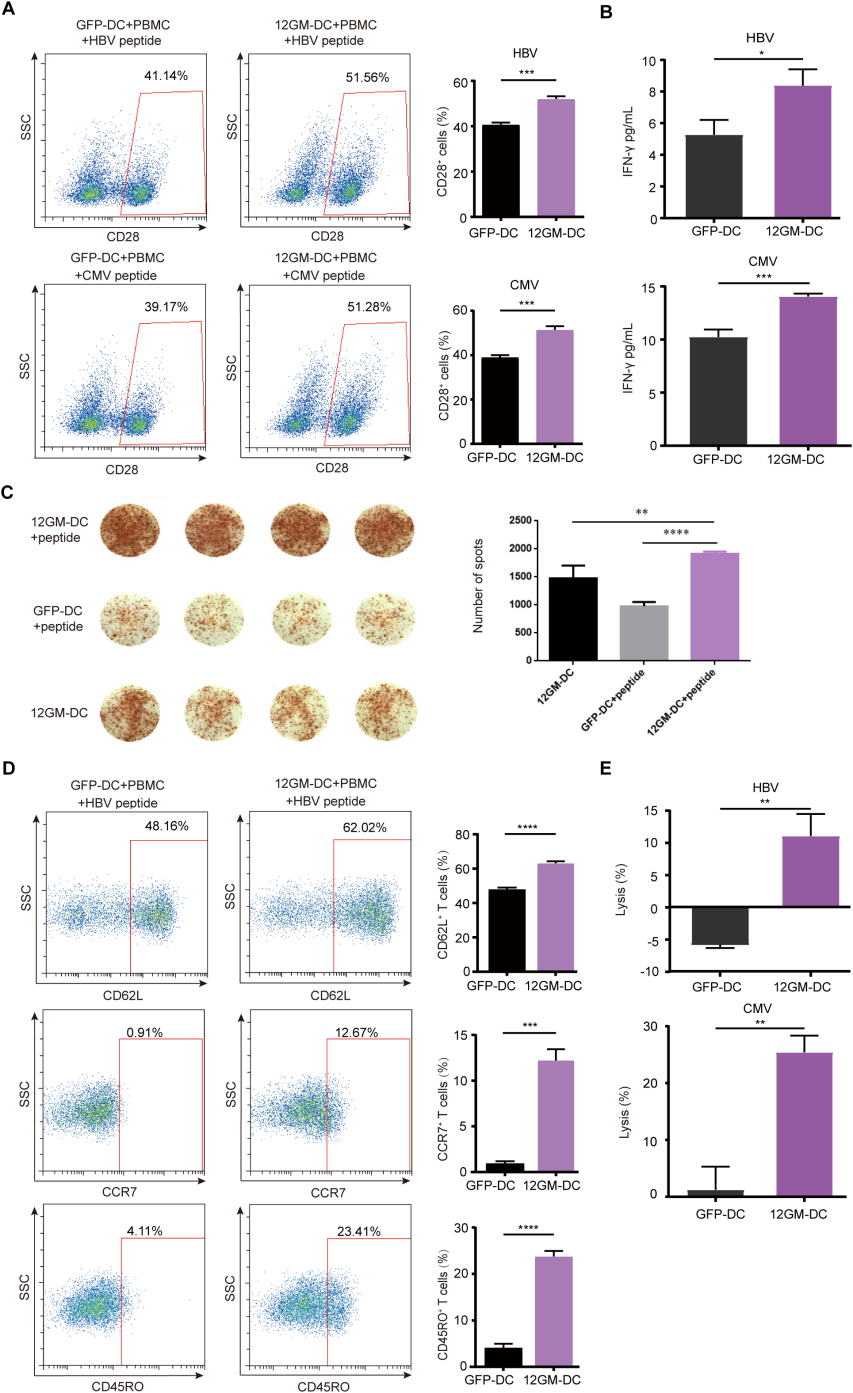
12GM-DCs optimize the enrichment of tumor virus-associated antigen-reactive T cells. PBMCs were co-cultured with HBV/CMV antigen-loaded 12GM-DCs for 1 week and then reactivated with antigens. **(A)** CD28 expression in CD3^+^ co-cultured cells was detected by flow cytometry. **(B)** IFN-γ secretion from T cells was measured by ELISA. **(C)** The IFN-γ positive spots secreted by PBMC after co-culture with DC loaded with HBV antigen were detected by ELISPOT assay. **(D)** The expression patterns of CD62L, CCR7, and CD45RO on T cells were detected by flow cytometry. **(E)** HBV/CMV antigen-loaded 12GM-DCs or GFP-DCs were co-cultured with 1G4 TCR-T cells for 1 week; target cells expressing the corresponding antigens were added at a target-to-effector ratio of 1:1. After 24 hours, the cytotoxicity of TCR-T cells toward target cells was measured using a luciferase chemiluminescence assay. Data are presented as the means ± SEMs. *P < 0.05; **P < 0.01; ***P < 0.001, ****P < 0.0001. The experiment was repeated at least three times.

After successfully generating 12GM-DCs that secrete high levels of IL-12 and GM-CSF and preliminarily verifying their ability to promote T cell function, we investigated potential applications in the *in vitro* enrichment and screening of antigen-reactive T cells. First, we assessed the impact of 12GM-DCs on the enrichment of T cells reactive to tumor virus-associated antigens. The viral antigens used in this experiment were 9-amino-acid peptides from Hepatitis B Virus (HBV) and Cytomegalovirus (CMV), both capable of binding to HLA-A*0201. T cell clones recognizing HBV and CMV antigens are widely present in human T cells. For this experiment, we used DCs induced by monocytes from HLA-A*0201-restricted donors and autologous PBMCs. 12GM-DCs loaded with HBV or CMV antigens were co-cultured with autologous PBMCs; GFP-DCs served as the control. After 6 days of co-culture, antigens were added again to the enriched and expanded T cells. Twenty-four hours later, T cells were analyzed. Compared with co-cultures containing GFP-DCs, the proportion of CD28-positive antigen-reactive T cells and the number of IFN-γ spots positive antigen-reactive T cells was significantly higher in co-cultures with 12GM-DCs (**Figures 2A, C**); the average concentration of secreted IFN-γ also was significantly higher in co-cultures with 12GM-DCs (**Figure 2B**). These findings suggest that 12GM-DCs enhance the activation of HBV/CMV antigen-reactive T cells and promote IFN-γ secretion. Furthermore, the ability of 12GM-DCs to promote the activation and cytokine secretion in antigen-reactive T cells was superior to the effects achieved by direct addition of IL-12 and GM-CSF (**Supplementary Figures S2A, B**). Previous studies have shown that the addition of IL-12 *in vitro* can help maintain CD62L expression and promote the generation of central memory T cells (19). Thus, we investigated the effect of 12GM-DCs on the phenotype of tumor virus-associated antigen-reactive T cells. The results showed that the expression levels of CD62L, CCR7, and CD45RO in virus antigen-reactive T cells co-cultured with 12GM-DCs were significantly increased (**Figure 2D**, **Supplementary Figure S2C**), indicating that 12GM-DCs preserve the memory phenotype of these T cells. This effect was also superior to the results from the direct addition of IL-12 and GM-CSF (**Supplementary Figure S2D**).

To assess the impact of 12GM-DCs on the cytotoxicity of antigen-reactive T cells, we engineered COS7 cells to overexpress CD80, CD83, CD86, and human HLA-A*0201 (20, 21), along with luciferase as a marker for target cells (**Supplementary Figures S3A-C**). After 6 days of T cell enrichment culture, target cells and peptides were added to these T cells. Biological luminescence was measured 24 hours later. The results showed that 12GM-DCs exhibited significantly enhanced cytotoxicity toward target cells (**Figure 2E**). In summary, 12GM-DCs effectively enriched and expanded tumor virus-associated antigen-specific T cells, enhancing their activation, cytokine secretion, and specific cytotoxicity. Therefore, 12GM-DCs can optimize the *in vitro* enrichment of tumor virus-associated antigen-reactive T cells.

### 3.3 12GM-DCs optimize the enrichment of TAA-reactive T cells

**Figure 3:**
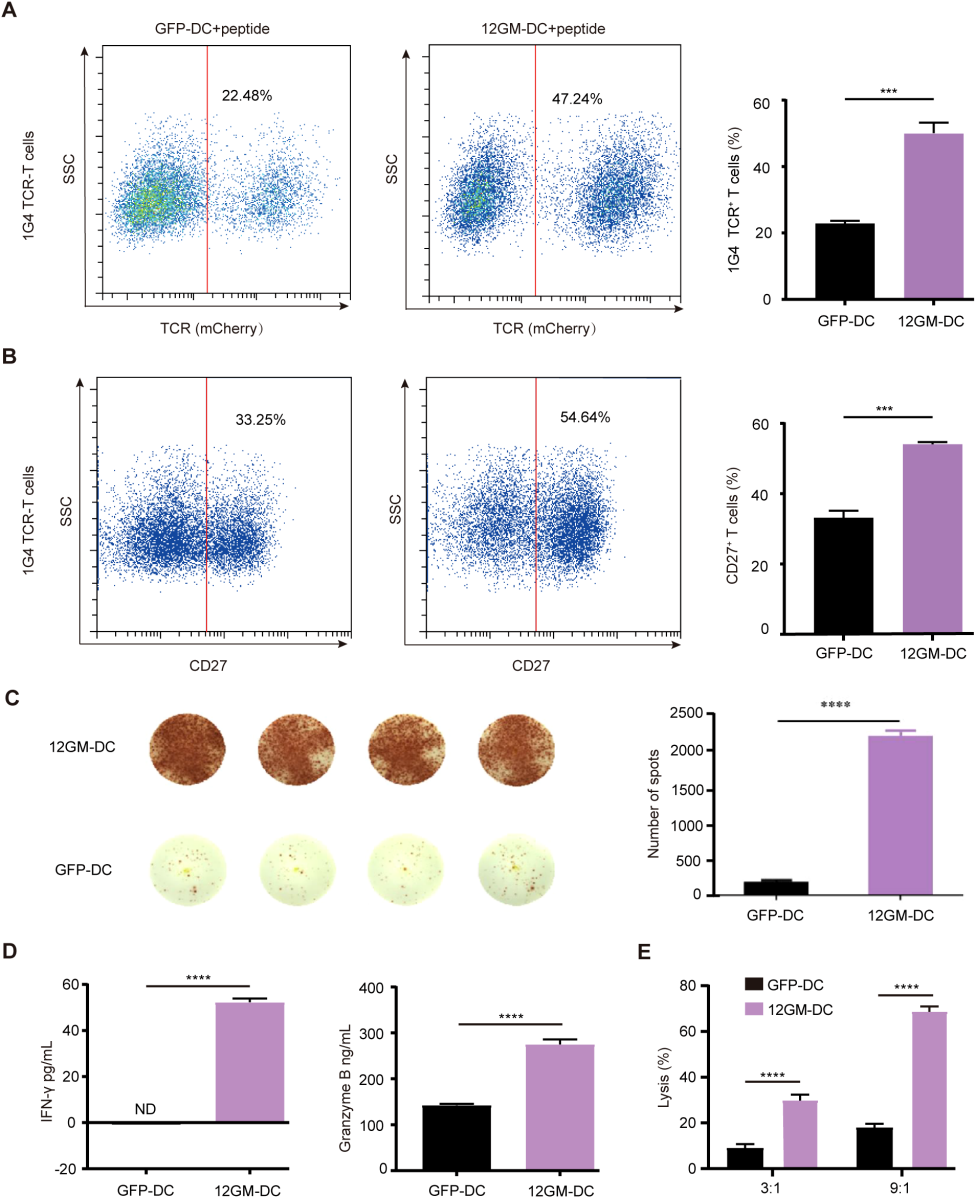
12GM-DCs optimize the enrichment of TAA-reactive T cells. 12GM-DCs or GFP-DCs were loaded with NY-ESO-1 antigen and then co-cultured with 1G4 TCR-T cells for 1 week. **(A)** The expression patterns of exogenous TCR on T cells were detected by flow cytometry. **(B)** CD27 expression on CD3^+^ co-cultured cells was detected by flow cytometry. **(C)** The IFN-γ positive spots secreted by PBMC after co-culture with DC loaded with NY-ESO-1 antigen were detected by ELISPOT assay. **(D)** Levels of IFN-γ and granzyme B secretion from T cells were measured by ELISA assay. **(E)** NY-ESO-1 antigen-loaded 12GM-DCs or GFP-DCs were co-cultured with 1G4 TCR-T cells for 1 week; target cells expressing the corresponding antigen were added at target-to-effector ratios of 3:1 and 9:1, respectively. After 24 hours, the cytotoxicity of TCR-T cells toward target cells was measured using a luciferase chemiluminescence assay. Data are presented as the means ± SEMs. ***P < 0.001, ****P < 0.0001. The experiment was repeated at least three times.

For the next experiment, we utilized 1G4 TCR-T cells, which recognize NY-ESO-1, as TAA-reactive T cells in an ex vivo enrichment and screening strategy. The NY-ESO-1 antigen used in this experiment was a 9-amino acid peptide that binds to HLA-A*0201. 1G4 TCR-T cells were generated through lentiviral transduction (**Supplementary Figure S3D**). The proportion of 1G4 TCR-T cells in the overall T cell population was assessed by flow cytometry. The results showed that after co-culture with 12GM-DCs, the proportion of 1G4 TCR-T cells had approximately doubled (**Figure 3A**). This finding indicates that 12GM-DCs specifically amplified NY-ESO-1-reactive T cells, increasing the population of these specific T cells.

Subsequently, we investigated the impact of 12GM-DCs on the activation of TAA-reactive T cells. Relative to co-cultures containing GFP-DCs, the proportion of CD27-positive TCR-T cells was significantly higher in co-cultures with 12GM-DCs (**Figure 3B**), at the same time, the number of IFN-γ spots positive antigen-reactive T cells in 12GM-DC group was higher (**Figure 3C**). Additionally, the average concentrations of IFN-γ and granzyme B in 12GM-DC-enriched TCR-T cells were significantly increased (**Figure 3D**), indicating that 12GM-DCs enhanced both activation and cytokine secretion in TAA-reactive T cells. We then explored the effect of 12GM-DCs on the cytotoxicity of tumor antigen-reactive T cells. NY-ESO-1 target cells (**Supplementary Figures S3B, C**) were co-cultured with T cells—subjected to 6 days of enrichment—at various ratios (T cells vs. target cells, 3:1 and 9:1). The results demonstrated that TCR-T cells enriched by co-culture with GFP-DCs exhibited minimal cytotoxicity against target cells, whereas co-culture with 12GM-DCs significantly increased cytotoxicity toward target cells (**Figure 3E**). Meanwhile, through *in vitro* experiments, we ruled out the allo-reaction of 1G4-T cells (**Supplementary Figure S3E**). These findings suggest that 12GM-DCs enhance the cytotoxicity of TAA-reactive T cells. This enhancement could be attributed to either an improvement in reactive T cell function or an increase in the number of antigen-reactive T cells. Overall, 12GM-DCs effectively optimized the ex vivo enrichment of TAA-reactive T cells.

### 3.4 12GM-DCs optimize the enrichment of neoantigen-reactive T cells

**Figure 4:**
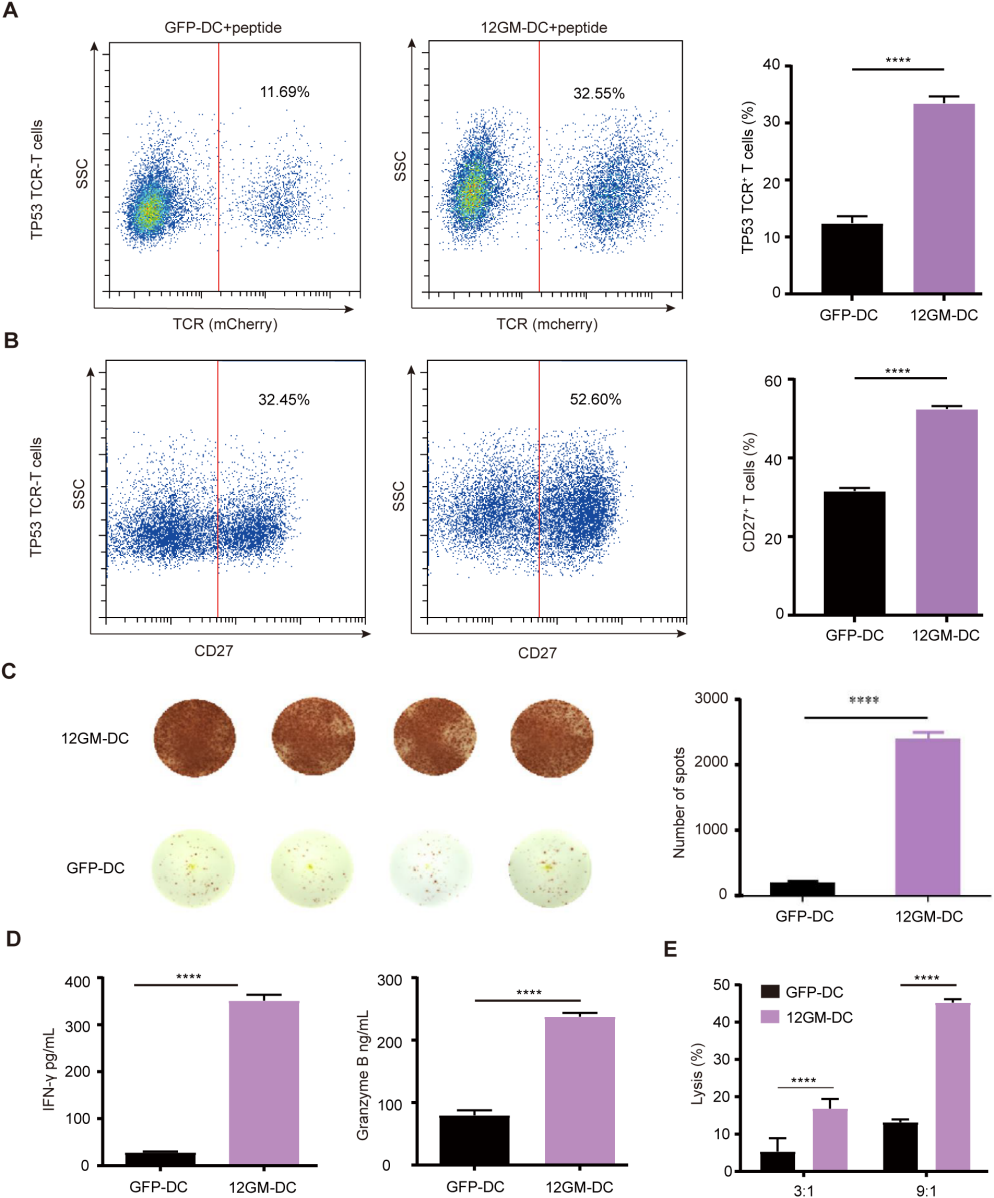
12GM-DCs optimize the enrichment of neoantigen-reactive T cells. 12GM-DCs or GFP-DCs were loaded with TP53 antigen and then co-cultured with TP53 TCR-T cells for 1 week. **(A)** The expression patterns of exogenous TCR on T cells were detected by flow cytometry. **(B)** CD27 expression on CD3^+^ co-cultured cells was detected by flow cytometry. **(C)** The IFN-γ positive spots secreted by PBMC after co-culture with DC loaded with TP53 antigen were detected by ELISPOT assay. **(D)** Levels of IFN-γ and granzyme B secretion from T cells were measured by ELISA. **(E)** TP53 antigen-loaded 12GM-DCs or GFP-DCs were co-cultured with TP53 TCR-T cells for 1 week; target cells expressing the corresponding antigen were added at target-to-effector ratios of 3:1 and 9:1, respectively. After 24 hours, the cytotoxicity of TCR-T cells toward target cells was measured using a luciferase chemiluminescence assay. Data are presented as the means ± SEMs. ****P < 0.0001. The experiment was repeated at least three times.

Tumor heterogeneity is driven by factors such as genetic mutations, epigenetic changes, and the tumor microenvironment (22, 23). Neoantigens are a subset of tumor-specific antigens that arise from mutations in tumor cells. These mutations can cause the immune system to recognize neoantigens as foreign, abnormal proteins expressed only in tumor cells (24, 25). This specificity increases the potential for a strong immune response against tumor cells, reducing the risk of damage to normal tissues and minimizing the potential for immune tolerance and autoimmunity (26). Here, we used TP53 (R175H, HMTEVVRHC) tumor mutant antigen-specific TP53 TCR-T cells as a model for the ex vivo enrichment and screening of neoantigen-reactive T cells. After co-culture with 12GM-DCs, the proportion of TP53 TCR-T cells nearly tripled from 11.69% (GFP control) to 32.55% (**Figure 4A**). Additionally, the proportion of CD27-positive cells was significantly increased (**Figure 4B**), at the same time, the number of IFN-γ spots positive antigen-reactive T cells in 12GM-DC group was higher (**Figure 4C**). And the average concentrations of IFN-γ and granzyme B were significantly higher (**Figure 4D**). These results demonstrate that 12GM-DCs effectively enriched antigen-specific TCR-T cells, enhancing activation and cytokine secretion in neoantigen-reactive TCR-T cells. Additionally, relative to co-cultures containing GFP-DCs, neoantigen-reactive T cells enriched through co-culture with 12GM-DCs exhibited significantly greater cytotoxicity against target cells (**Figure 4E**). In summary, 12GM-DCs optimize the ex vivo enrichment and screening of neoantigen-reactive T cells.

### 3.5 12GM-DCs optimize the enrichment of individualized neoantigen-reactive T cells

Tumor heterogeneity is mainly reflected in inter-patient variability, intra-tumor diversity, and differences between tumors (1). Neoantigens are highly specific to individual patients, suggesting that personalized treatment can be achieved by targeting tumor-reactive T cells specific to these neoantigens. In this study, tumor-reactive T cells were screened using individualized mutant neoantigens from tumor patients. Samples were obtained from two colorectal cancer patients CRC-1 (HLA-A*03:01; HLA-A*02:07) and CRC-2 (HLA-A*24:02; HLA-A*30:01), and individualized neoantigen-reactive T cell screening was performed (**Supplementary Figure S4A**). For patient CRC-1, 24 peptides were synthesized, whereas 63 peptides were synthesized for patient CRC-2 (**Table 1**; **Additional file 2**). Compared with the dimethyl sulfoxide control group, a higher proportion of CD137-positive T cells was detected in all peptide groups (**Supplementary Figures S4B, D**). Single T cells were then sorted by flow cytometry for TCR sequencing to identify high-frequency TCRs. The results showed that peptide groups 1 and 3 for patient CRC-1 had high-frequency TCRs (**Supplementary Figure S4C**); similar results were observed in peptide groups 2 and 3 for patient CRC-2 (**Supplementary Figure S4E**).

**Table 1:** Personalized peptides for cancer patients.

Mutant gene	Neo-peptide	HLA	Icore	BindLevel
CRC-1				
ASB2-2	ARFDVNMPLA	HLA-B*27:05	ARFDVNMPLA	SB
ASB2-3	LSARFDVNMPL	HLA-B*27:05	ARFDVNMPL	SB
CGB2-2	KRLLLLLLLSM	HLA-B*27:05	KRLLLLLLL	SB
FKRP-1	ARQPPLATAL	HLA-B*27:05	ARQPPLATAL	SB
FKRP-2	RQPPLATALAR	HLA-C*02:02	RQPPLATAL	WB
KMO-2	LAISSTYLL	HLA-C*02:02	LAISSTYLL	SB
KMO-3	LLAISSTYL	HLA-C*02:02	LAISSTYL	WB
KMO-1	SLLAISSTY	HLA-A*03:01	SLLAISSTY	WB
MYO9B-1	RRRPTSSFVTVR	HLA-B*27:05	RRPTSSFVTVR	SB
MYO9B-2	RRRTSSFVTVR	HLA-B*27:05	RRTSSFVTV	SB
MYO9B-3	RTSSFVTVRVK	HLA-A*03:01	RTSSFVTVR	SB
MYO9B-5	RRRPTSSFVTV	HLA-B*27:05	RRRPTSSFV	WB
MYO9B-6	TSSFVTVRVK	HLA-A*03:01	SSFVTVRVK	SB
MYO9B-7	RRTSSFVTVRV	HLA-B*27:05	RRTSSFVTV	SB
MYO9B-9	RRPTSSFVTVRV	HLA-B*27:05	RRPTSSFVTVR	SB
SNAPC4-1	QRCWRHALHR	HLA-B*27:05	QRCWRHALHR	SB
SNAPC4-2	RCWRHALHRRL	HLA-B*27:05	WRHALHRRL	SB
ASB2-1	SARFDVNMPL	HLA-B*27:05	ARFDVNMPL	SB
FKRP-3	RQPPLATAL	HLA-C*02:02	RQPPLATAL	WB
MMP25	QRFAGLPETDR	HLA-B*27:05	QRFAGLPETDR	SB
MYO9B-4	RRRTSSFVTV	HLA-B*27:05	RRTSSFVTV	SB
MYO9B-8	TSSFVTVRVK	HLA-A*03:01	SSFVTVRVK	SB
CGB2-1	SKRLLLLLLL	HLA-B*27:05	KRLLLLLLL	SB
CGB2-3	MSKRLLLLLLL	HLA-B*27:05	KRLLLLLLL	SB
CRC-2				
ANKS1B	LAAQDYRYY	HLA-C*12:02	LAAQDYRYY	SB
APOB	MTKQSFDLSVK	HLA-A*30:01	MTKQSFDLSVK	SB
CGNL1	LVHSRKEEK	HLA-A*30:01	LVHSRKEEK	SB
CIAO3	TTRSPQQVM	HLA-A*30:01	TTRSPQQVM	SB
DLG2	AQKYGRLQV	HLA-A*30:01	AQKYGRLQV	SB
MTR	ASRSVVVCS	HLA-A*30:01	ASRSVVVCS	WB
LILRB5	RTLWAEPASV	HLA-B*13:02	TLWAEPASV	SB
PCDH15	RIQAALPTAK	HLA-A*30:01	RIQAALPTAK	SB
OR51G1-1	MSIDRYVAI	HLA-C*12:02	MSIDRYVAI	SB
PALB2-2	IWNLKPGQLL	HLA-A*24:02	IWNLKPGQLL	SB
HCAR3	VVRVHIFWL	HLA-A*30:01	VVRVHIFWL	WB
GRID1	FVMFYNSEY	HLA-C*12:02	FVMFYNSEY	SB
PALB2-1	NLKPGQLLK	HLA-A*30:01	NLKPGQLLK	SB
NUP155	ATRAFCRYGG	HLA-A*30:01	ATRAFCRYG	WB
RAET1G	KFRPGPRWCVV	HLA-A*30:01	KFRPGPRWC	WB
RYR1	KFAEYTHENW	HLA-A*24:02	KFAEYTHENW	SB
EFHC2	ASHYRVPEG	HLA-A*30:01	ASHYRVPEG	
FCGBP	FVASGGACM	HLA-C*12:02	FVASGGACM	SB
FLG2	GSRQSSGYG	HLA-A*30:01	GSRQSSGYG	
MARCHF7	SSMVRGSFG	HLA-A*30:01	SSMVRGSF	WB
OR51G1-2	RYVAICNPL	HLA-A*24:02	RYVAICNPL	WB
PLCG1	REIKEICPGK	HLA-A*30:01	REIKEICPGK	WB
PLXNA4-1	IYLGAINRI	HLA-A*24:02	IYLGAINRI	SB
PPP1R8	KWISTLTI	HLA-A*24:02	KWISTLTI	WB
ZNF831-2	LQAETPLPL	HLA-C*12:02	LQAETPLPL	WB
PLXNA4-2	MYLGAVNRI	HLA-A*24:02	MYLGAVNRI	SB
PLXNA4-3	RTGHMYLGA	HLA-A*30:01	RTGHMYLGA	WB
PTPRG-1	RVRLRPLPGK	HLA-A*30:01	RVRLRPLPGK	SB
PTPRG-2	HSRVRLRPL	HLA-A*30:01	HSRVRLRPL	WB
PTPRG-3	LAYDHSRVRL	HLA-C*12:02	LAYDHSRVRL	SB
RAD17-1	RSLVREYSTSI	HLA-B*13:02	LVREYSTSI	WB
SPEG	SYMSAGEEPL	HLA-A*24:02	SYMSAGEEPL	WB
TMEM60-1	FTTMILSYV	HLA-C*12:02	FTTMILSY	WB
TMEM60-3	QFTTMILSYVF	HLA-A*24:02	TTMILSYVF	WB
TMEM60-2	TTMILSYVF	HLA-A*24:02	TTMILSYVF	WB
TSHZ3-2	WHQSAMAKM	HLA-C*06:02	WHQSAMAKM	
ZBTB37-1	RFQAILNQHF	HLA-A*24:02	RFQAILNQHF	SB
SCN11A-1	RVKVIVGALL	HLA-A30:01	RVKVIVGAL	SB
TTI1	LVNDEIPEI	HLA-C*12:02	LVNDEIPEI	WB
ZBTB37-2	CGKSFRFQA	HLA-A*30:01	CGKSFRFQA	
ZNF831-1	KYLLRLLQA	HLA-A*30:01	KYLLRLLQA	SB
SCN11A-2	VSRVKVIVGA	HLA-A*30:01	RVKVIVGA	WB
ZDHHC15	MFFGSLVILF	HLA-A*24:02	FFGSLVILF	SB
ZNF681-1	RSSHLTRHK	HLA-A*30:01	RSSHLTRHK	SB
ADAMTS12-1	RSLSWRSISK	HLA-A*30:01	RSLSWRSISK	SB
ADCK5	RGWSRLASA	HLA-A*30:01	RGWSRLASA	WB
CBFA2T2	ATNFPLCPFV	HLA-A*12:02	ATNFPLCPF	
CCDC172	DYMESVYEAL	HLA-A*24:02	DYMESVYEAL	WB
CLCN5	MAADMTSKW	HLA-C*12:02	MAADMTSKW	SB
CNOT1	ITTPGSFAL	HLA-C*12:02	ITTPGSFAL	SB
DRC1	RLRIAACLEA	HLA-A*30:01	RLRIAACLEA	
ERVFRD-1	KTKGPNQSQT	HLA-A*30:01	KTKGPNQSQ	SB
ESR1	RKDQRGGRMLK	HLA-A*30:01	KDQRGGRMLK	SB
ABCA1	GSIELPHYVK	HLA-B*13:02	GSIELPHYV	SB
ADAMTS12-2	SRSLSWRSI	HLA-C*06:02	SRSLSWRSI	SB
PTN	RALHNAKCQK	HLA-A30:01	ALHNAKCQK	SB
RAD17-2	GDWNTRSLV	HLA-B*13:02	GDWNTRSLV	WB
TSHZ3-1	AMAKMLQQV	HLA-B*13:02	AMAKMLQQV	SB
RASAL3	GSRESLSTL	HLA-A*30:01	GSRESLSTL	SB
SH2D3A	LLIPQVSRL	HLA-C*12:02	LLIPQVSRL	WB
U2AF2	GAKNATLLS	HLA-A*30:01	GAKNATLLS	
ZNF419	RSCKVHPSEK	HLA-A*30:01	RSCKVHPSEK	SB
ZNF681-2	KAFNRSSHLTR	HLA-A*12:02	KAFNRSSHL	SB

The underlined parts represent mutated amino acids.

**Figure 5:**
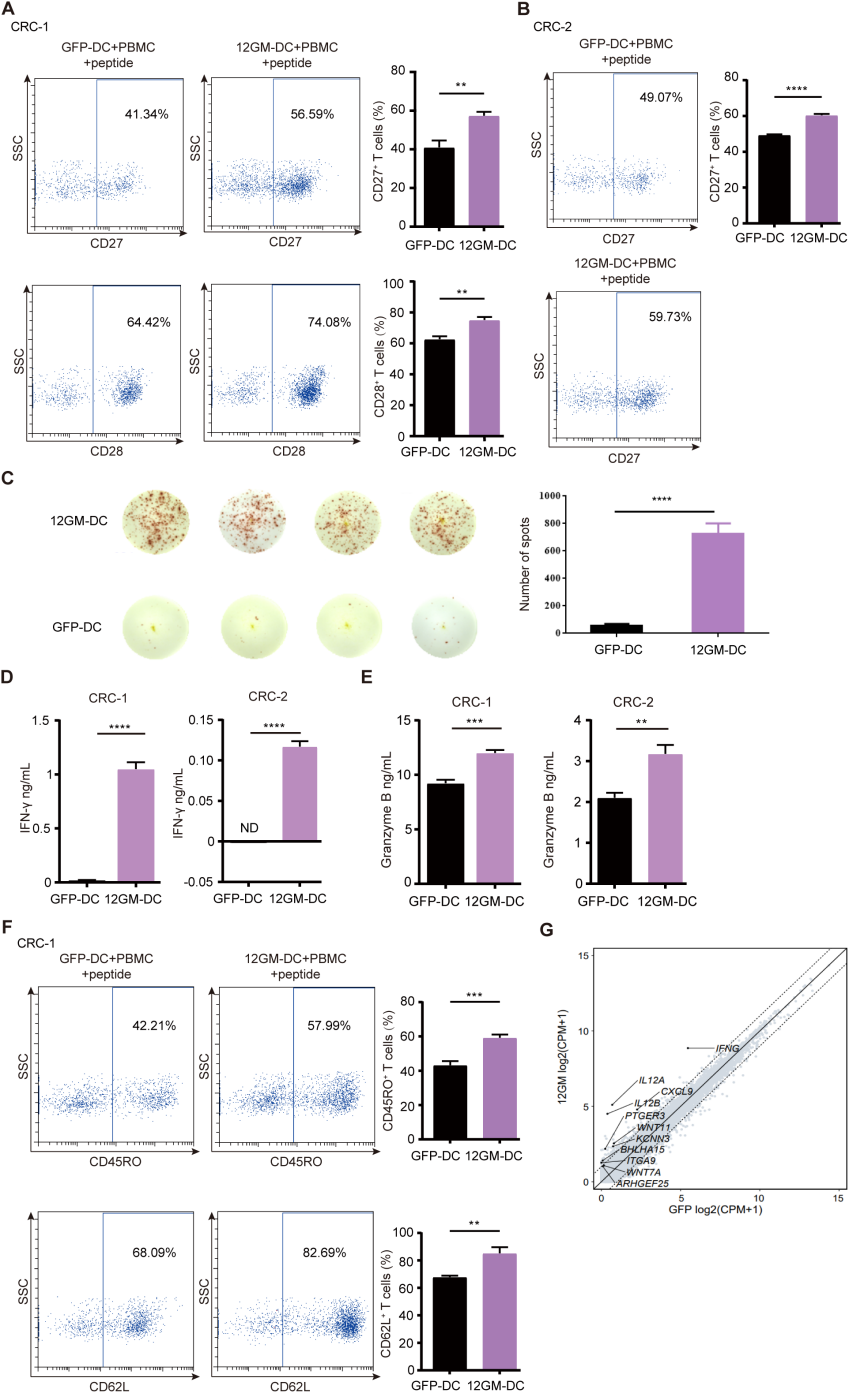
The role of 12GM-DCs in the enrichment of individualized neoantigen-reactive T cells. 12GM-DCs or GFP-DCs loaded with individualized neoantigens from patients CRC-1 and CRC-2 patients were co-cultured with PBMCs for 1 week, then reactivated by antigen stimulation for 18 hours. **(A, B)** CD27 and CD28 expression patterns CD3^+^ on co-cultured cells were detected by flow cytometry. **(C)** The IFN-γ positive spots secreted by PBMC after co-culture with DC loaded with individualized neoantigens from patients CRC-1 were detected by ELISPOT assay. **(D)** IFN-γ secretion from T cells was measured by ELISA. **(E)** Granzyme B secretion from T cells was measured by ELISA. **(F)** CD62L and CD45RO expression patterns on T cells were detected by flow cytometry. **(G)** The GO diagram for the upregulated pathways in the co-cultured cells transcriptome was analyzed. Data are presented as the means ± SEMs. **P < 0.01; ***P < 0.001, ****P < 0.0001. The experiment was repeated at least three times.

Building on the experimental system established earlier in this study, we explored the role of 12GM-DCs in optimizing the *in vitro* enrichment and screening of individualized neoantigen-reactive T cells from tumor patients. The results showed significant upregulation of CD27 and CD28 in CRC-1 neoantigen-reactive T cells enriched by 12GM-DCs (**Figure 5A**). We confirmed these findings in the CRC-2 sample, which revealed consistent results (**Figure 5B**). At the same time, the number of IFN-γ spots positive antigen-reactive T cells in 12GM-DC group was higher (**Figure 5C**). Compared with co-cultures containing GFP-DCs, the average concentrations of IFN-γ and granzyme B in neoantigen-reactive T cells from patients CRC-1 and CRC-2, co-cultured with 12GM-DCs, were significantly higher (**Figures 5D, E**); the expression levels of CD62L and CD45RO also were significantly upregulated (**Figure 5F**). Meanwhile, we conducted RNA sequencing analysis on the co-cultured cells (**Figure 5G**). Collectively, our findings indicate that 12GM-DCs enhance the activation, cytokine secretion, and memory phenotype of neoantigen-reactive T cells from colorectal cancer patients. Thus, 12GM-DCs can effectively optimize the *in vitro* enrichment and screening of individualized neoantigen-reactive T cells from tumor patients.

### 3.6 The 12GM-DC culture system sustains T cell phenotypes and promotes anti-tumor effect of tumor antigen-reactive T cells *in vivo*

**Figure 6:**
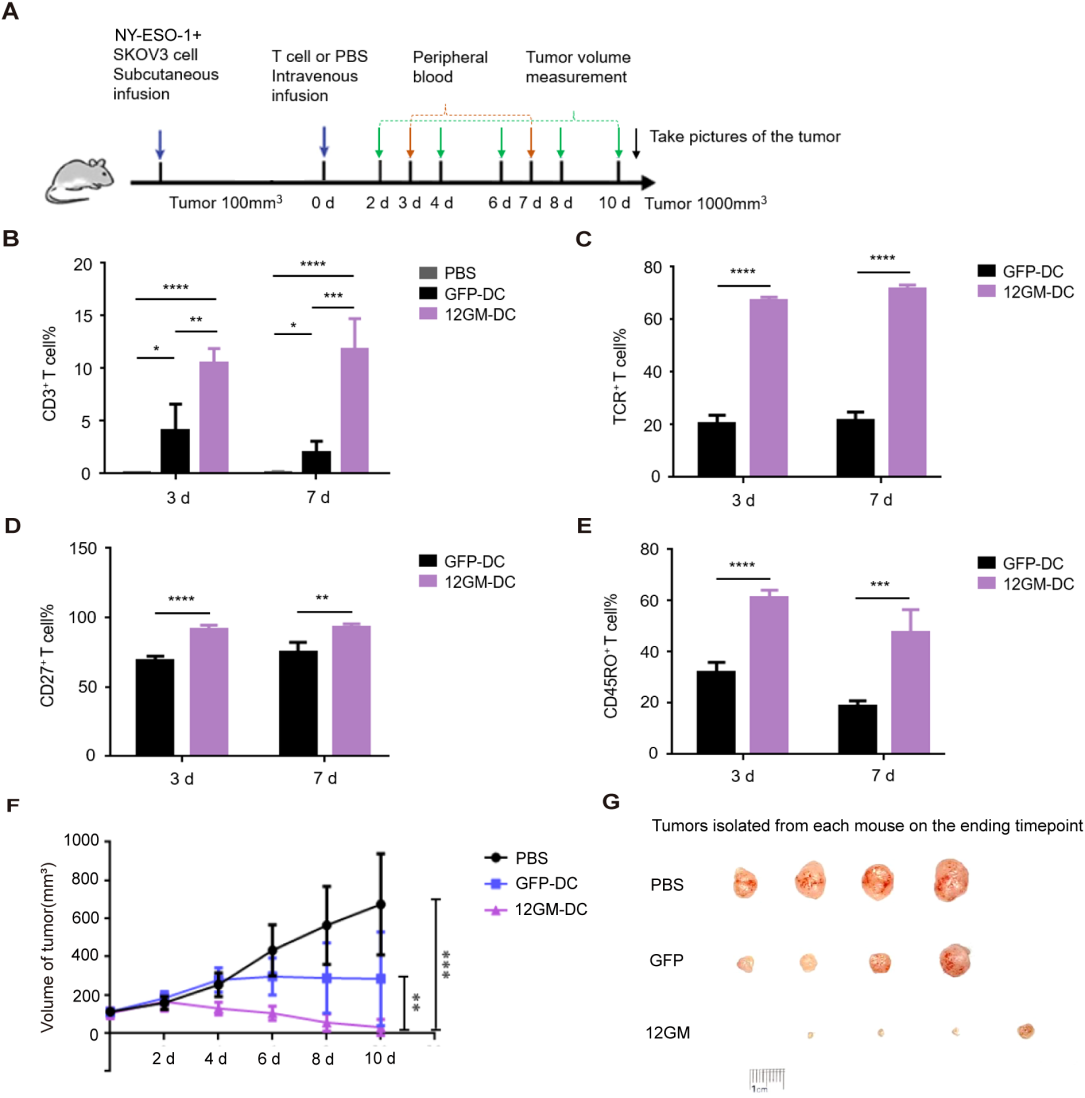
The 12GM-DC culture system promotes the anti-tumor effect of tumor antigen-reactive T cells *in vivo*. **(A)** A schematic of the *in vivo* antitumor activity of tumor antigen-reactive T cells in 12GM-DC culture system. PBS group, n=4; GFP-DC group n=4; 12GM-DC group, n=5. **(B)** The maintenance of human CD3^+^ T cells in peripheral blood of mice was detected by flow cytometry. **(C)** The expression of exogenous TCR in human CD3^+^ T cells in peripheral blood of mice was detected by flow cytometry. **(D)** The expression of CD27 in human CD3^+^ T cells in peripheral blood of mice was detected by flow cytometry. **(E)** The expression of CD45RO in human CD3^+^ T cells in peripheral blood of mice was detected by flow cytometry. **(F)** Plot of changes in tumor growth in mice after infusion of tumor antigen-reactive T cells in the 12GM-DC culture system. **(G)** Images of mouse tumors after treatment with tumor antigen-reactive T cells in the 12GM-DC culture system. Mice were sacrificed by cervical dislocation method when the tumor volume approached 1000 mm^3^, and tumors were removed for photography. Data are presented as the means ± SEMs. *P < 0.05; **P < 0.01; ***P < 0.001, ****P < 0.0001.

To investigate whether reactive T cells in the 12GM-DC culture system can still exhibit phenotypic and functional advantages *in vivo*, we stablished a tumor model in immunodeficient mice xenograft with SKOV3 cells (HLA-A*02:01^+^). Mice were divided into different groups according to tumor size and each group was administrated with PBS, 1G4 TCR-T cells cultured in GFP-DC or 12GM-DC culture system, and continuously monitored for tumor growth and T cells in peripheral blood (**Figure 6A**). We designed the experiment not to isolate the T cells and inject the mixed culture (**Supplementary Figure S5A**).

We collected the peripheral blood and analyzed T cell number sustainance and functional phenotypes from different groups on the 3rd and 7th days after infusion. We found that the percentage of human CD3^+^ T cells in the peripheral blood of mice with tumor antigen-reactive T cells cultured in 12GM-DC culture system was significantly higher than that of GFP-DC group and maintained for at least 7 days (**Figure 6B**, **Supplementary Figure S5C**). The positive rate of exogenous TCR expression in human CD3^+^ T cells in 12GM-DC group was significantly higher than that in GFP-DC group and maintained for at least 7 days (**Figure 6C**, **Supplementary Figure S5D**). At the same time, antigen-specific T cells in 12GM-DC group were further enriched and expanded *in vivo* compared with that before infusion. These results indicated that 12GM-DC culture system could promote the maintenance of antigen-specific T cells in tumor antigen-reactive T cells *in vivo*. Further, we detected the expression of functional T phenotypes. The percentage of CD27 positive tumor antigen-reactive T cells in 12GM-DC group was significantly higher than that in GFP-DC group (**Figure 6D**, **Supplementary Figure S5E**), indicating that 12GM-DC culture system enhanced the activation of tumor antigen-reactive T cells *in vivo*. At the same time, the expression of CD45RO in human CD3^+^ T cells in peripheral blood of mice in 12GM-DC group was significantly up-regulated (**Figure 6E**, **Supplementary Figure S5F**), indicating that 12GM-DC culture system promoted the maintenance of memory phenotype of tumor antigen-reactive T cells *in vivo*.

In the meantime, we detected the tumor volume changes every two days to find whether 12GM-DC culture T cells possess enhanced anti-tumor activity. We found that tumor growth was inhibited to a certain extent in GFP-DC group. Strikingly, tumor growth was effectively inhibited in 12GM-DC group, which was significantly less than the other two groups (**Figures 6F, G**). Meanwhile, through *in vitro* experiments, we ruled out the allo-reaction of 1G4-T cells (**Supplementary Figure S5B**). At the same time, we also sequenced the HLA subtypes of the infused T cells (**Supplementary Table S1**). Since the HLA compatibility is unknown, the alloreactive responses cannot be fully ruled out. Therefore, all these data from *in vivo* tumor-burden mouse indicate that 12GM-DC culture system promoted the anti-tumor activity of tumor antigen-reactive T cells and significantly inhibited tumor growth *in vivo*.

## 4 Discussion

In this study, we genetically modified DCs to overexpress IL-12 and GM-CSF to generate 12GM-DCs, then developed a 12GM-DC-based culture system to optimize the activation and proliferation of tumor-reactive T cells. In this improved culture system, 12GM-DCs were able to enrich reactive T cells in the presence of various tumor antigens, including virus-associated tumor antigens, tumor-associated antigens, tumor-mutated neoantigens, and individualized neoantigens from tumor patients. Additionally, 12GM-DCs increased the proportion of antigen-specific T cells, enhanced the activation of reactive T cells, and promoted the maintenance of a memory phenotype in these T cells. 12GM-DCs also enhanced the secretion of IFN-γ and granzyme B, leading to improved specific cytotoxicity in antigen-reactive T cells. Notably, 12GM-DC culture system sustained T cell functional phenotypes and promoted the anti-tumor activity of tumor antigen-reactive T cells to greatly inhibit tumor growth *in vivo*. Thus, we have developed a novel tumor-reactive T cell culture system for efficient enrichment and expansion of antigen-specific T cells.

Our study achieved in the enhancement of the activation and enrichment of tumor-reactive T cells through combining the expressing IL-12 and GM-CSF in DCs. Conventional antigen-reactive TCR-T development has been greatly hindered by the dysfunctional state often exhibited by tumor-reactive T cells (27, 28). Considering the enhanced immune efficacy of IL-12 and GM-CSF, we hypothesized that expressing these cytokines in dendritic cells could improve the quality of tumor-reactive T cells. In a previous study, an oncolytic adenovirus expressing IL-12 and GM-CSF, combined with a DC vaccine, exhibited robust anti-tumor efficacy (29). It has also been demonstrated that antigen-presenting cells secreting GM-CSF can induce cytotoxic T cell-mediated anti-tumor responses (30). Additionally, several investigations have revealed that IL-12 promotes antigen-reactive T cell activation, along with secretion of IFN-γ from those cells (12, 13, 18). Based on our findings that 12GM-DCs enhance the activation of reactive T cells while promoting the maintenance of a memory phenotype, we have shown expanded roles for these two cytokines in regulating tumor-reactive T cells. This advancement could facilitate the enrichment and screening of neoantigen-specific T cells for improved cancer immunotherapy.

Our newly developed 12GM-DC tumor-reactive T cell culture system holds great potential for advancing the use of tumor antigens and their reactive T cells in combating solid tumors. Given that the current accuracy of antigen prediction is less than 50% (31), effective verification of the clinical relevance of predicted tumor epitopes remains a major challenge in the clinical application of antigen-reactive T cells. Although tetramers can simultaneously recognize multiple antigen-specific T cells, their synthesis is complex and costly, and they are limited to specific major histocompatibility complex types (32, 33). Activation markers such as CD137 are commonly used to detect reactive T cells, but they have low sensitivity (34). These limitations must be addressed to enable broader application of antigen-reactive T cells in clinical cancer therapy. In the present study, we were able to obtain antigen-reactive T cells that could be effectively activated and expanded, thereby overcoming the limitation of dysfunctional tumor-reactive T cells during ex vivo enrichment and screening. 12GM-DCs enhanced antigen activation of T cells within the *in vitro* stimulation system; these activated antigen-reactive T cells expanded efficiently. In this study, tumor-reactive T cells were co-cultured with 12GM-DCs for 6 days, and the reactive T cells were enriched by 2 to 3 times. If the culture time was further extended, the enrichment effect would be even more significant. We successfully utilized 12GM-DCs to effectively enrich tumor-reactive T cells from two patients in our clinic, and we were able to detect a higher proportion of activated and more potent reactive T cells. These interactions enabled 12GM-DCs to enhance both the proportion and activation of antigen-specific T cells, allowing efficient selection and screening of this small but crucial subset of antigen-reactive T cells.

Our 12GM-DC tumor-reactive T cell culture system can also be applied to the expansion of tumor-reactive T cells, including tumor-infiltrating lymphocytes, which have shown therapeutic potential in the management of solid tumors. A key challenge in this application involves whether tumor-specific antigen-reactive T cells can be selectively amplified and enriched; this capability determines the efficacy of optimal tumor control. Our 12GM-DCs specifically expand antigen-specific T cells and enhance their secretion of IFN-γ and granzyme B, resulting in a higher proportion of tumor-killing T cells in the enriched culture. Furthermore, our *in vivo* data indicate that the memory phenotype of these cells was preserved, leading to stronger anti-tumor activity *in vivo*. Concurrently, the functional and phenotypic advantages of tumor antigen-reactive T cells derived from the 12GM-DC culture system could be also effectively maintained and the antitumor activity enhanced after infusion into mice. In conclusion, we developed an optimized culture system to enrich tumor antigen-specific T cells. This system has the potential to advance the clinical application of tumor immunotherapy by targeting tumor antigens and their reactive T cells.

Our study is one of a proof-of-concept culture system and many of improvements need to be explored in the future. As for future clinical application, DCs would be mainly added *in vitro* and will not be infused into the body, it is thus no severe clinical adverse reactions involved. Further, clinical-grade 12GM-DCs are required for developing culture system. The continuous secretion of IL-12 and GM-CSF may lead to a shift in T cell differentiation. IL-12 induces Th1 differentiation (35), while GM-CSF promotes Th17 differentiation (36). Meanwhile, both IL-12 and GM-CSF have been reported to cause an increase in inflammatory factors (28, 37), which may indirectly damage T cells. The continuous activation of T cells by IL-12 and GM-CSF may also cause metabolic stress in T cells. In this culture, the stimulation of T cells by 12GM-DCs within the system requires only a short-term time window. In future strategies, large-scale cell expansion becomes necessary, we propose replacing DCs with other APCs capable of long-term *in vitro* culture, as well as constructing vectors that enable sustained expression.

The 12GM-DC culture system can be further optimized by combining chemical small molecules, thereby developing a more superior *in vitro* culture system for screening tumor antigens and tumor-reactive T cells. Furthermore, this cultivation system can also be applied in the identification of the immunogenicity of a wide range of antigens, including viral antigens and autoantigens, etc.

## Data Availability

The original contributions presented in the study are included in the article/**Supplementary Material**. Further inquiries can be directed to the corresponding authors.
