# Supplementary material for: IL-12 and GM-CSF engineered dendritic cells enhance the enrichment and selection of tumor-reactive T cells for cancer immunotherapy
Source: Front Immunol. 2025 Nov 17;16:1684842. doi: 10.3389/fimmu.2025.1684842 (PMC12665776; doi:10.3389/fimmu.2025.1684842)
Supplement: Supplementary file 1 [file Table1.docx]

***Supplementary Material***
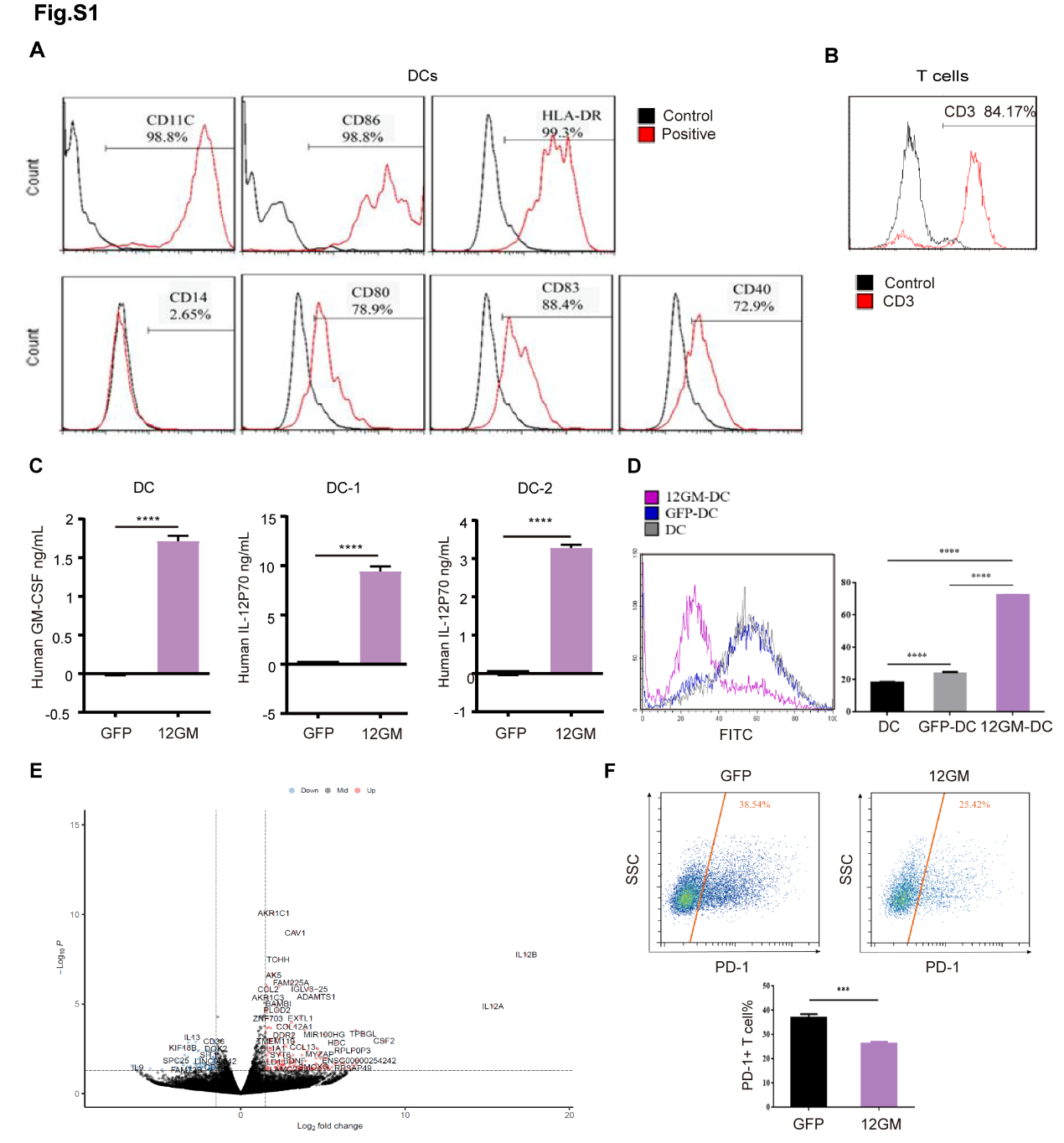


**Supplementary Figure 1.** A). The expression patterns of CD80, CD86, CD83, CD40, CD11C, CD14 and HLA-DR on cultured DCs were detected by flow cytometry. B). Purity of isolated T cells were detected by flow cytometry. C). After DCs had been infected with GFP or 12GM adenoviruses for 48 hours, levels of GM-CSF and IL-12 secretion were measured by ELISA. D). The proliferation of T cells after co-culture with GFP-DCs or 12GM-DCs was detected by CFSE method. E). The volcano plot of differentially expressed genes in the transcriptional profiles of GFP-DC and 12GM-DC 48 hours after adenovirus infection. F). CD28 expression on co-cultured cells was detected by flow cytometry. Data are presented as the means ± SEMs. ****P* < 0.001, *****P* < 0.0001.


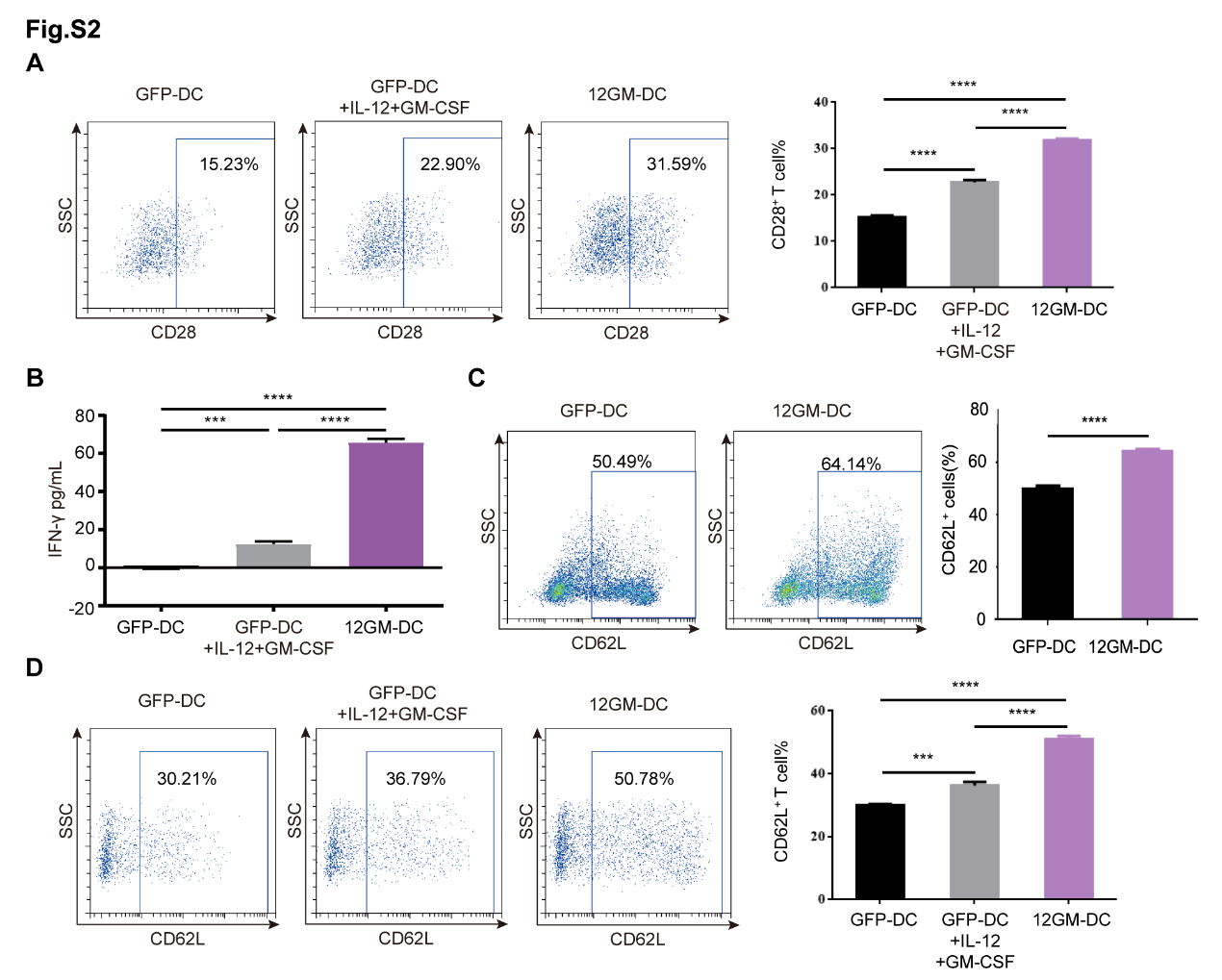


**Supplementary Figure 2.** A), B), D). 12GM-DCs, GFP-DCs, and GFP-DCs supplemented with 10 ng/mL IL-12 and 1000 IU/ml GM-CSF were co-cultured with PBMCs for 1 week, then reactivated by HBV antigen stimulation for 18 hours. A). CD28 expression on co-cultured cells was detected by flow cytometry. B). IFN-γ secretion from T cells was measured by ELISA. C). CMV antigen-loaded 12GM-DCs or GFP-DCs were co-cultured with PBMCs for 1 week, then reactivated by antigen stimulation for 18 hours. CD62L expression was detected by flow cytometry. D). CD62L expression on T cells was detected by flow cytometry. Data are presented as the means ± SEMs. ***P < 0.001, ****P < 0.0001.


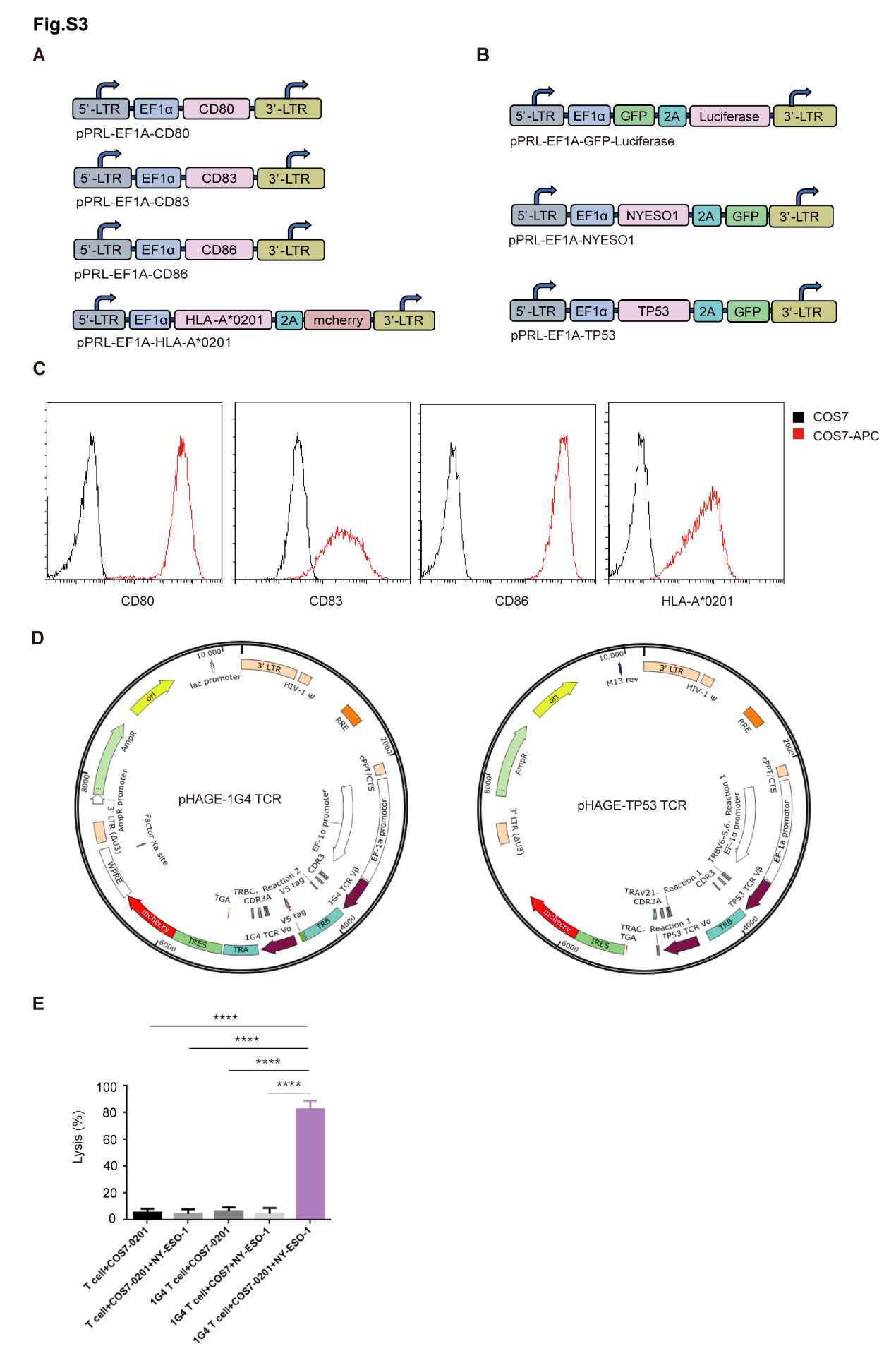


**Supplementary Figure 3.** A). Schematic diagrams of the structures of hCD80, hCD83, hCD86, and HLA-A*0201 lentiviruses. B). Schematic diagrams of the structures of NY-ESO-1, TP53, and luciferase lentiviruses. C). Expression patterns of overexpressed molecules in COS7 target cells were detected by flow cytometry. D). Schematic diagrams of the structures of 1G4 TCR and TP53 TCR lentiviruses. E). COS7 or COS7 -HLA-A*0201 cells and NY-ESO-1 antigen peptide were respectively added to the 1G4 TCR-T cells or the T cells; target cells were added at target-to-effector ratios of 3:1. After 24 hours, the cytotoxicity of T cell and TCR-T cells toward target cells was measured using a luciferase chemiluminescence assay. Data are presented as the means ± SEMs. ****P < 0.0001.


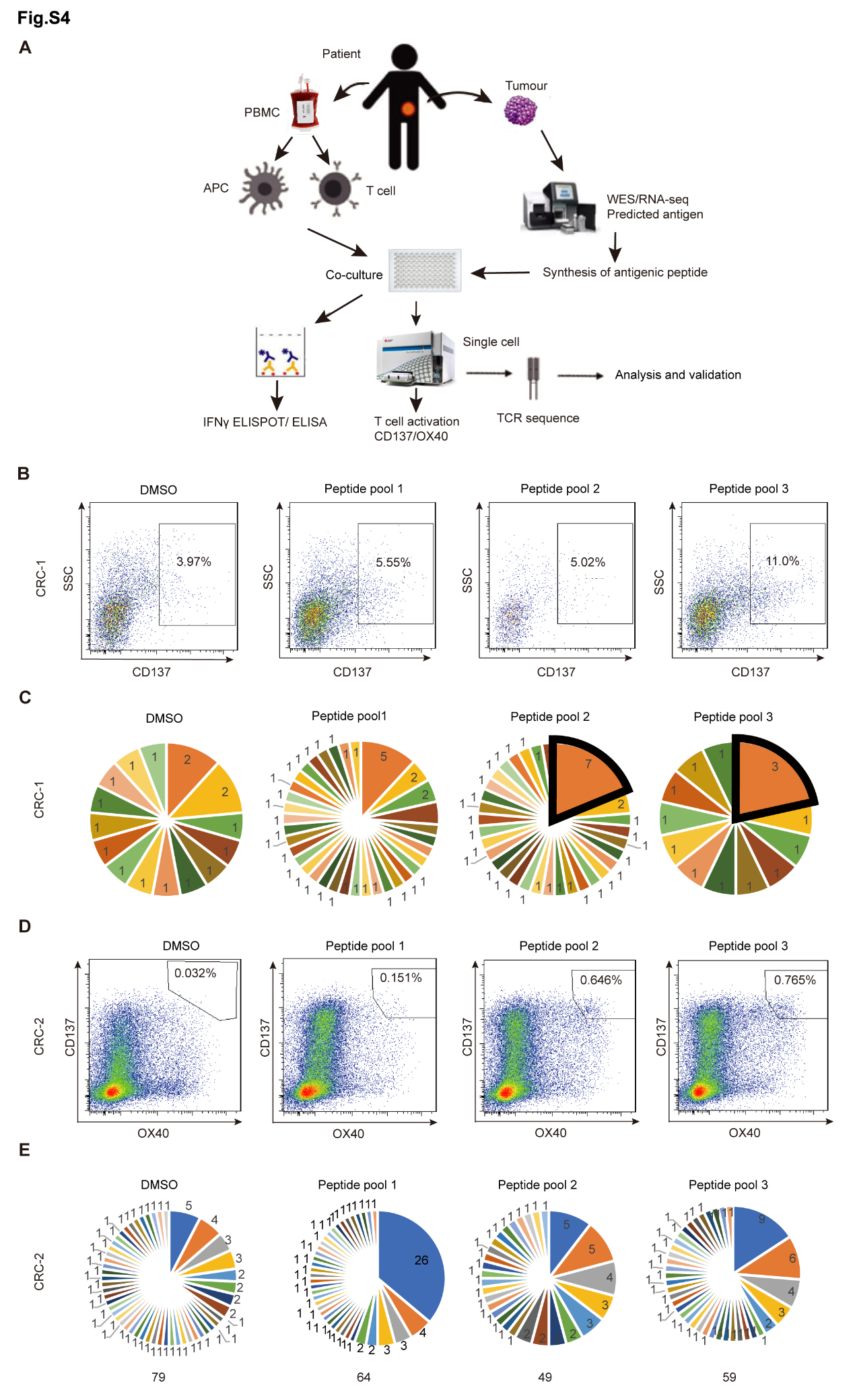


**Supplementary Figure 4.** A). Schematic diagram of screening for individualized neoantigen-reactive T cells from tumor patients. B). Screening for individualized neoantigen-reactive T cells in patient CRC-1. C). Analysis of individualized neoantigen-reactive TCRs in patient CRC-1. D). Screening for individualized neoantigen-reactive T cells in patient CRC-2. E). Analysis of individualized neoantigen-reactive TCRs in patient CRC-2.


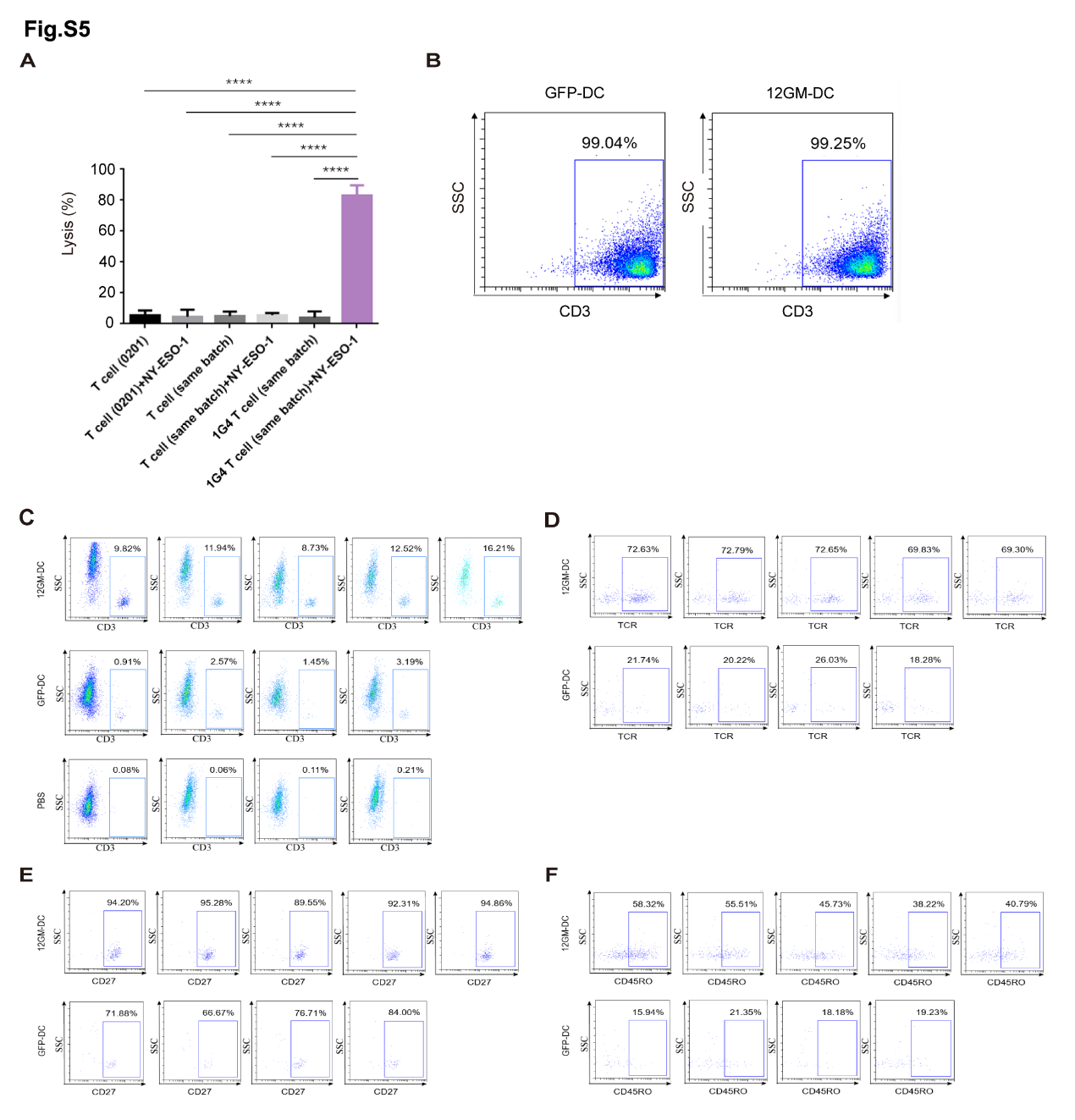


**Supplementary Figure 5.** A). SKOV-3 (HLA-A*0201) cells and NY-ESO-1 antigen peptide were respectively added to the T cells from the same batch in the in vivo experiment (non-HLA-A*0201) and the T cells with HLA-A*0201; target cells were added at target-to-effector ratios of 3:1. After 24 hours, the cytotoxicity of T cell and TCR-T cells toward target cells was measured using a luciferase chemiluminescence assay. B). Before infusing the co-cultured T cells into the mice, a flow cytometry test was conducted to examine the expression of CD3^+^ on the cell surface. C). The maintenance of human CD3^+^ T cells in peripheral blood of mice on the 7th day after infusion was detected by flow cytometry. D). The expression of exogenous TCR in human CD3^+^ T cells in peripheral blood of mice on the 7th day after infusion was detected by flow cytometry. E). The expression of CD27 in human CD3^+^ T cells in peripheral blood of mice on the 7th day after infusion was detected by flow cytometry. F). The expression of CD45RO in human CD3^+^ T cells in peripheral blood of mice on the 7th day after infusion was detected by flow cytometry. Data are presented as the means ± SEMs. ****P < 0.0001.

Supplementary Table 1 The HLA subtypes of the infused T cells

| **HLA-A** | **HLA-B** | **HLA-C** |  |
| --- | --- | --- | --- |
| HLA-A*24:02 | HLA-B*15:01 | HLA-C*04:01 |  |
| HLA-A*24:02 | HLA-B*40:01 | HLA-B*07:02 |  |
