# Supplementary figures and images for: IL-12 and GM-CSF engineered dendritic cells enhance the enrichment and selection of tumor-reactive T cells for cancer immunotherapy
Source: Front Immunol. 2025 Nov 17;16:1684842. doi: 10.3389/fimmu.2025.1684842 (PMC12665776; doi:10.3389/fimmu.2025.1684842)

***Supplementary Material***

The NetMHCpan - 4.1 Predicted neontigens


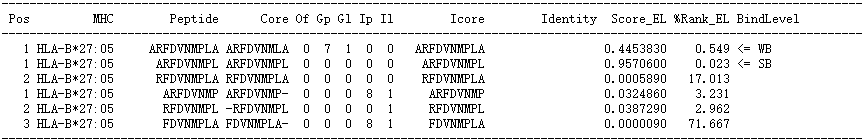

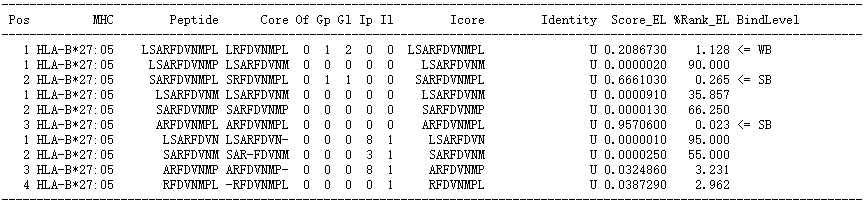

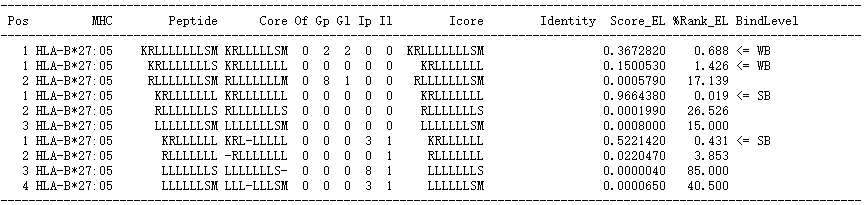

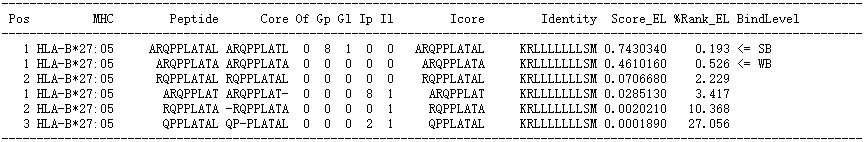

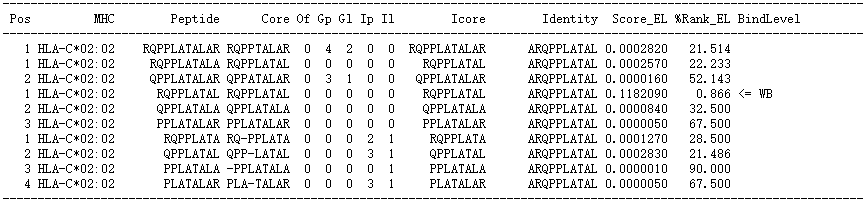

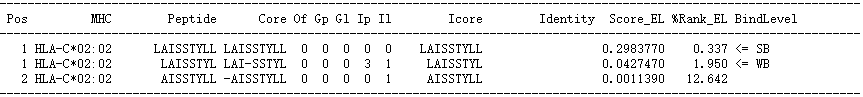

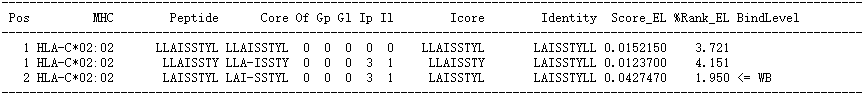

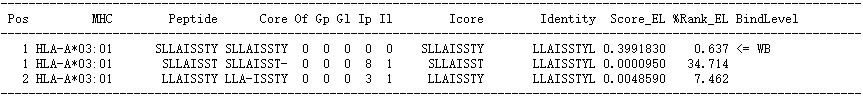

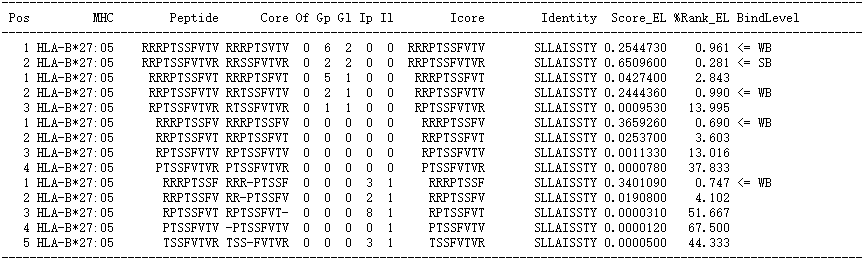

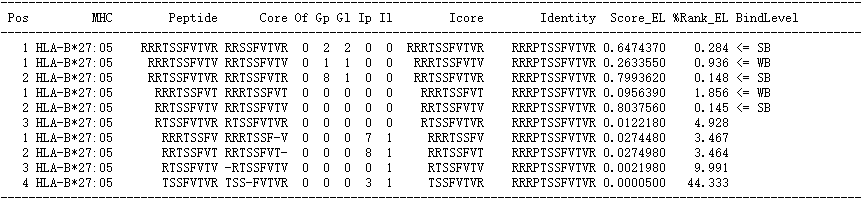

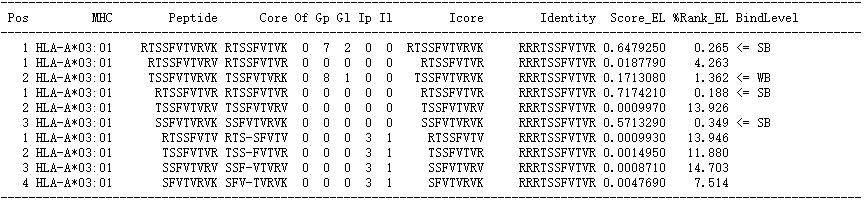

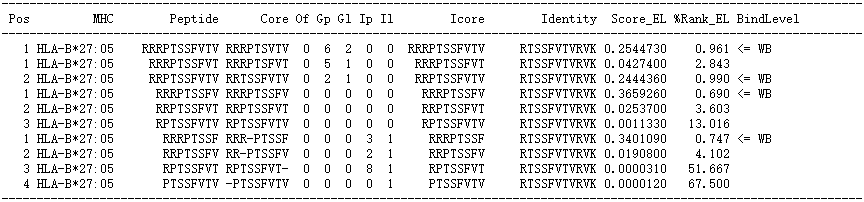

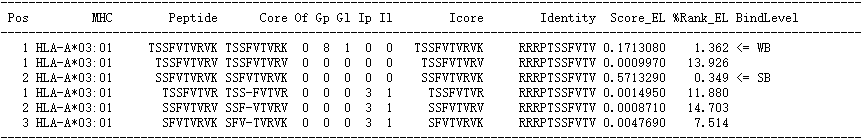

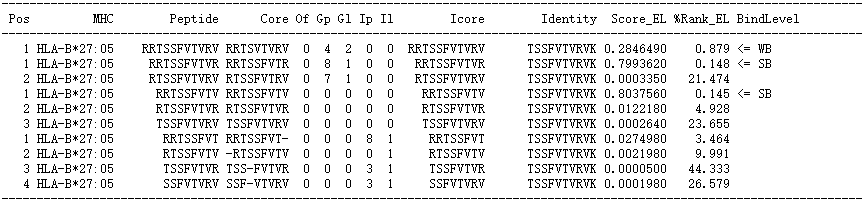

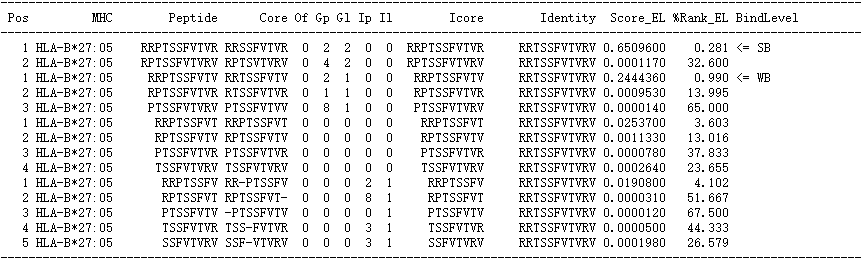

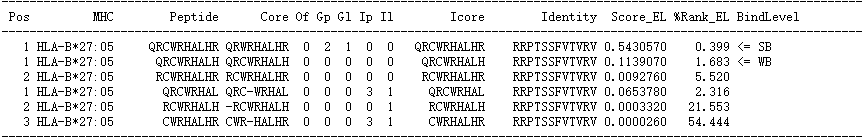

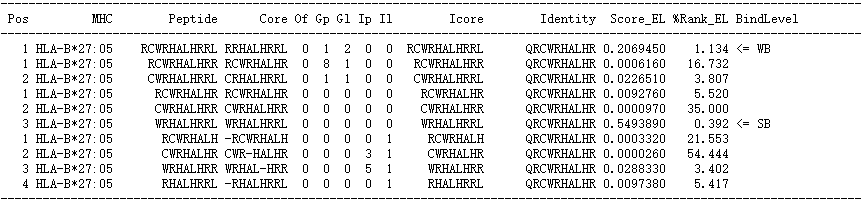

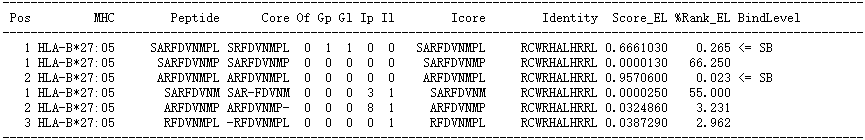

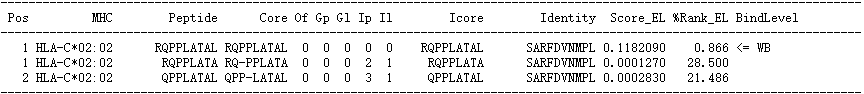

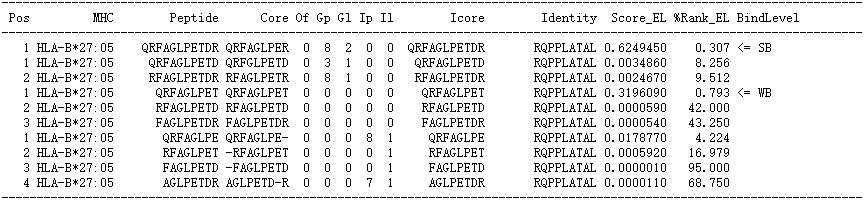

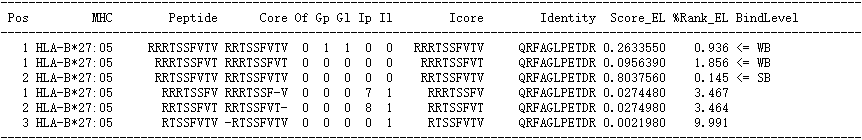

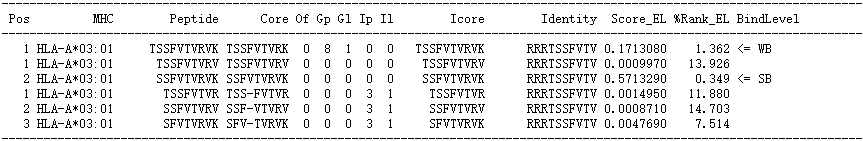

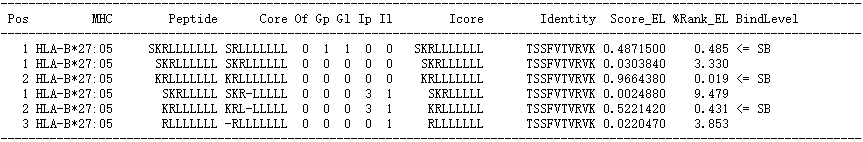

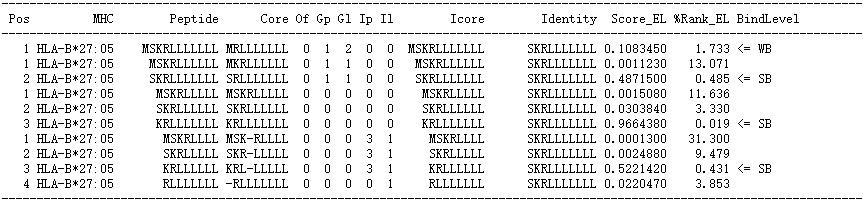


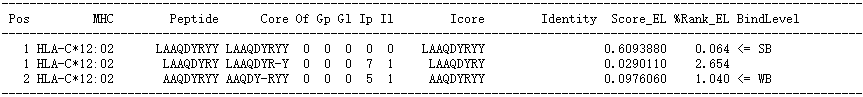

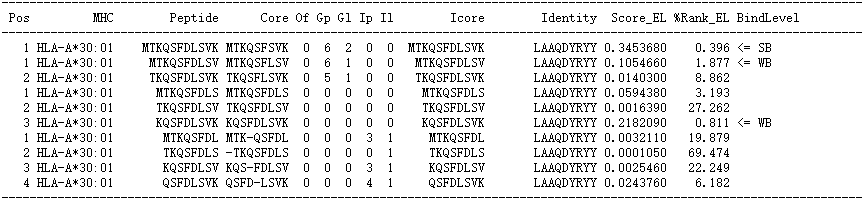
**
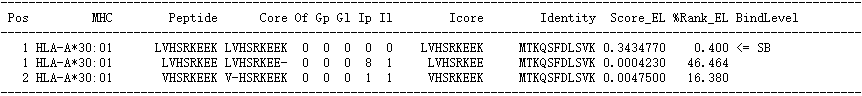
**
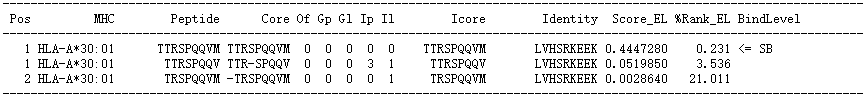
**
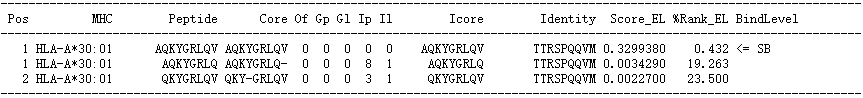

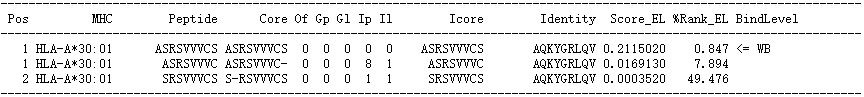

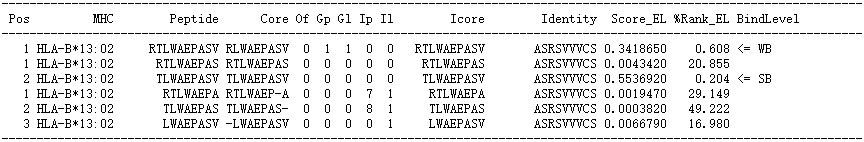
**
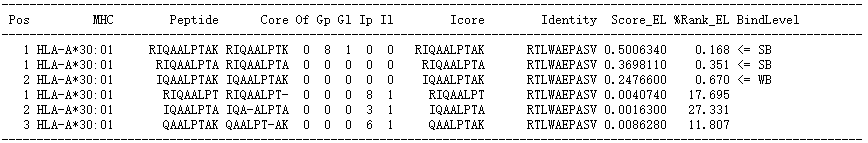

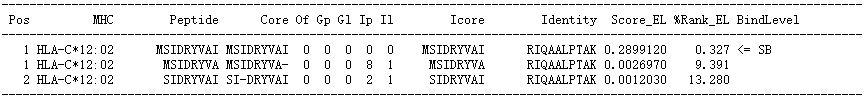
**
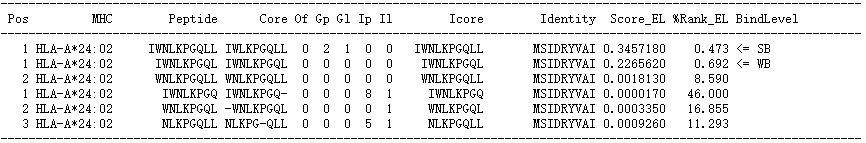
**
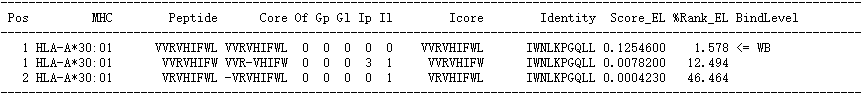

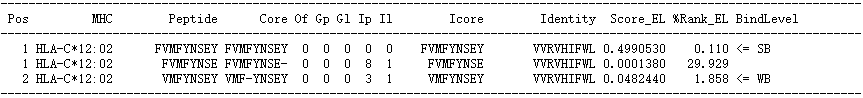

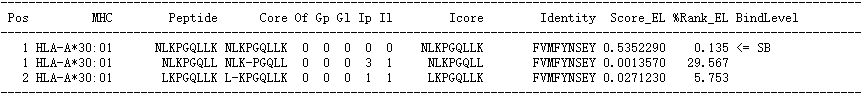

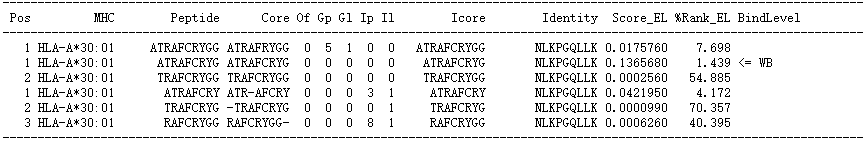
**
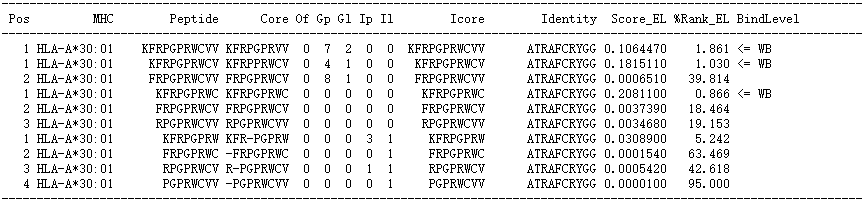
**
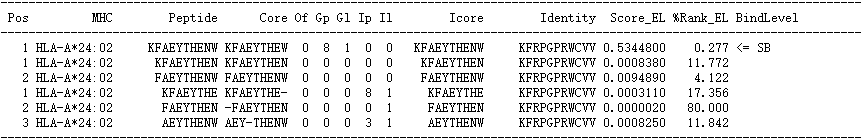
**
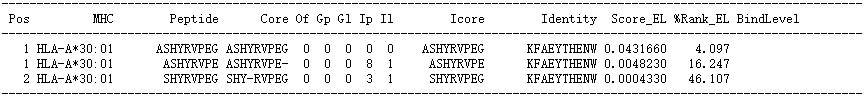
**
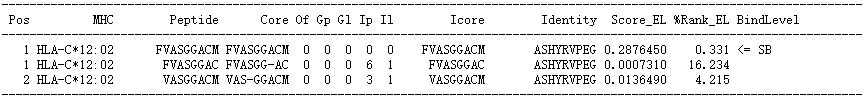

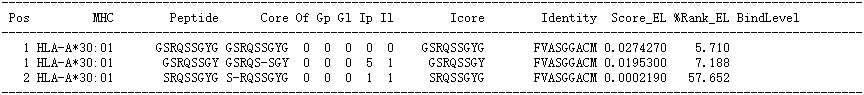

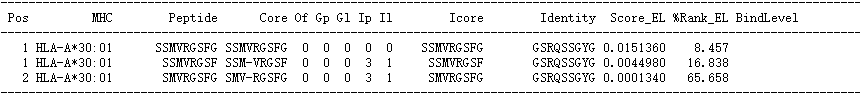

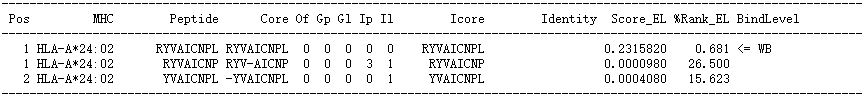


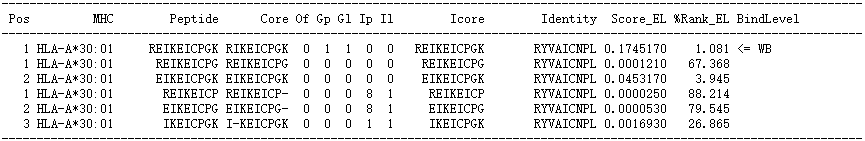
 **
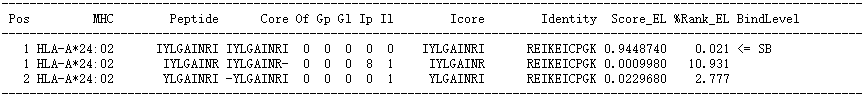
**
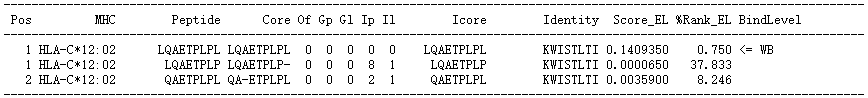

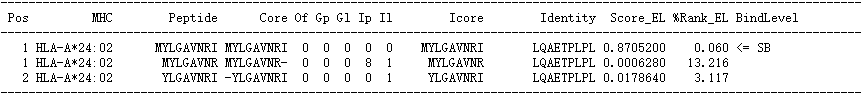

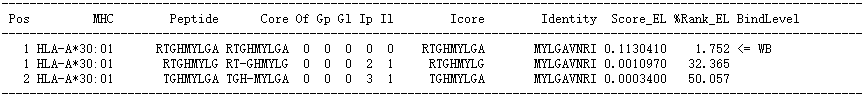

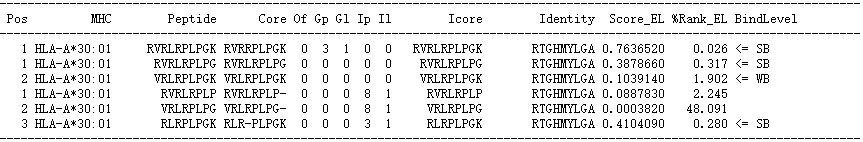

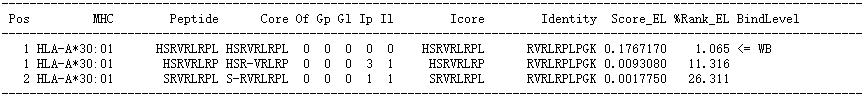


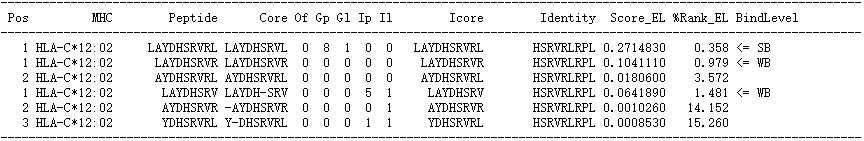
 **
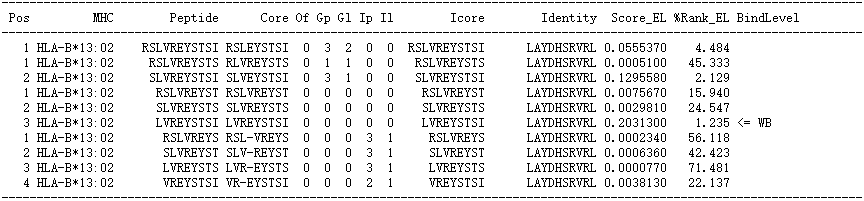
**

**
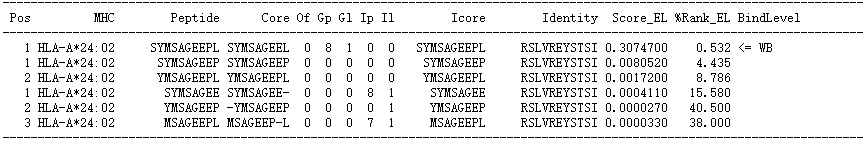
** **
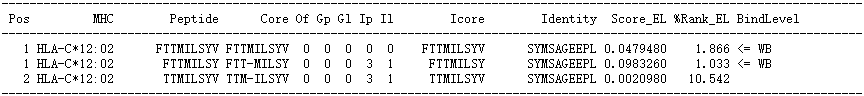
**


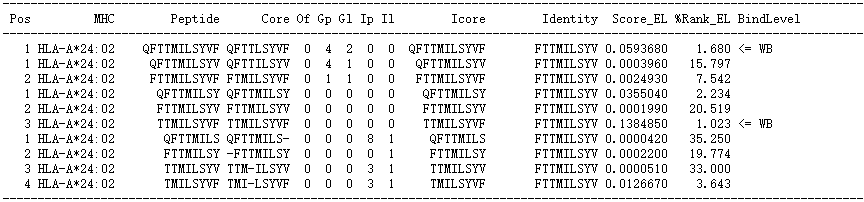

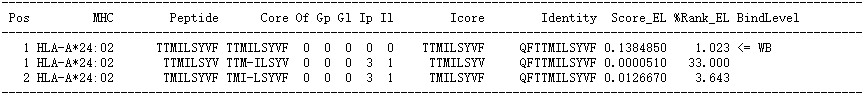

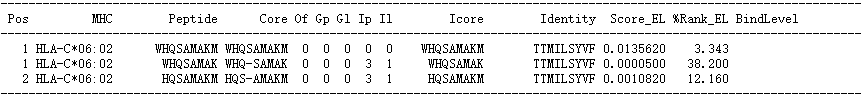

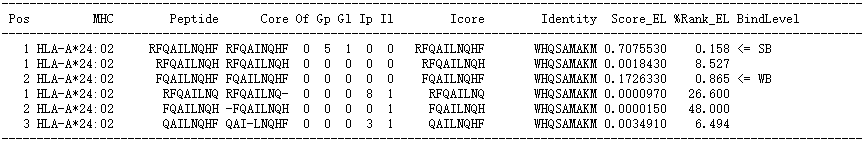


**
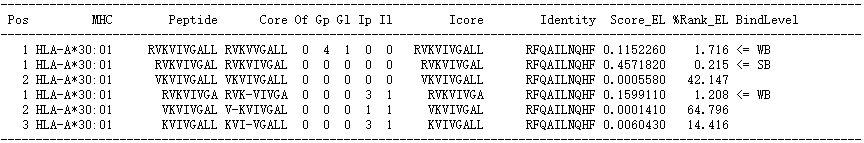
**
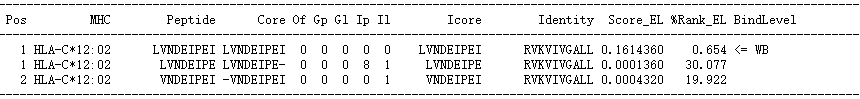

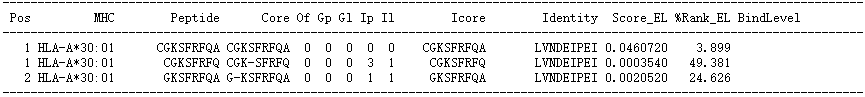

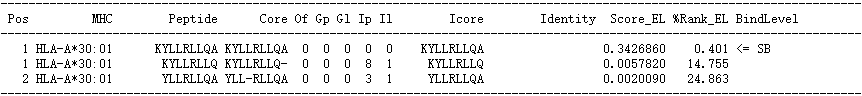

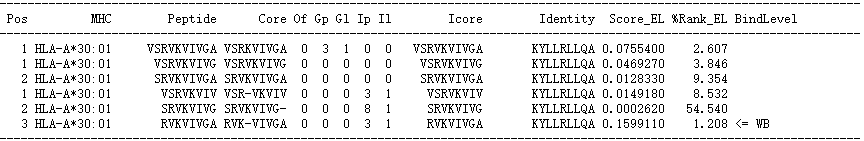

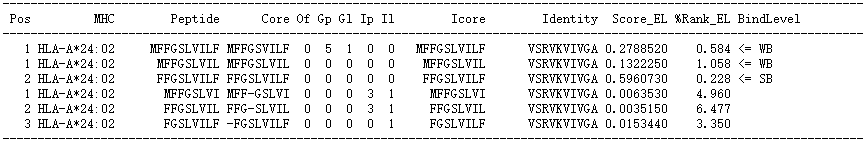

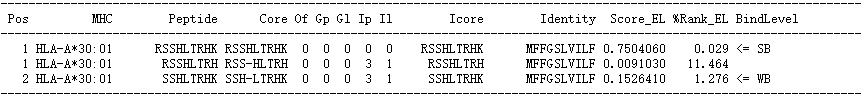

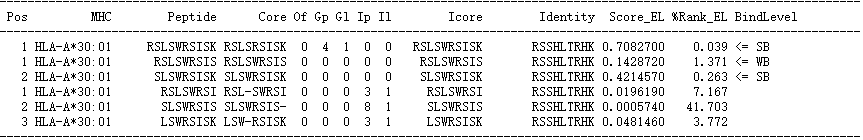

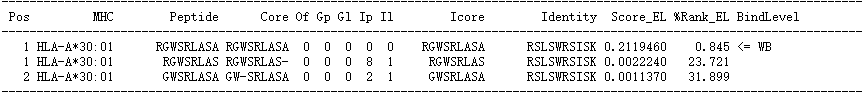

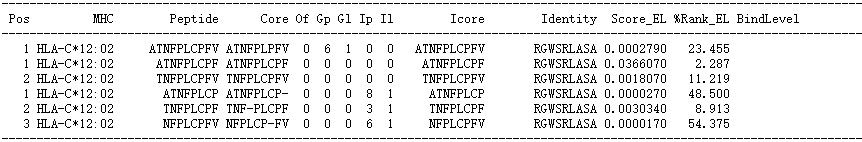

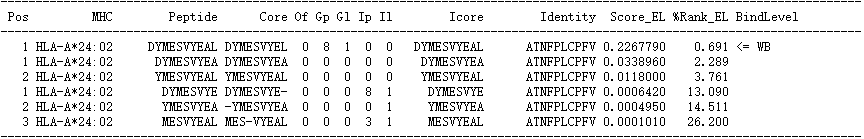

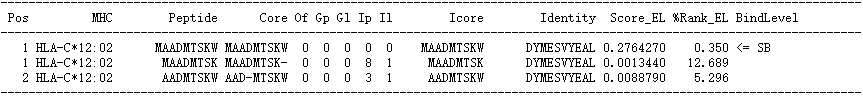

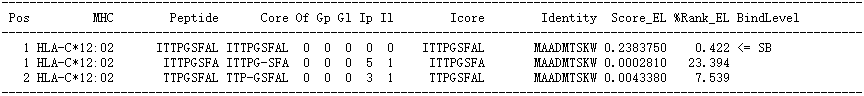


**
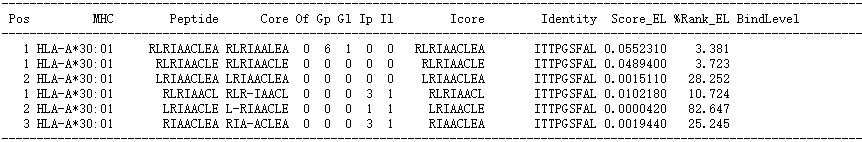
**
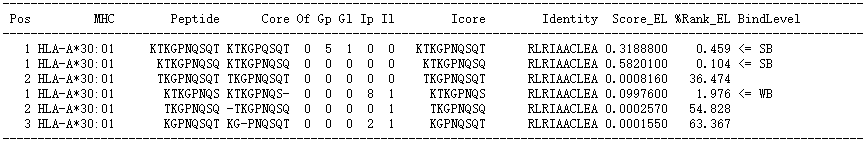

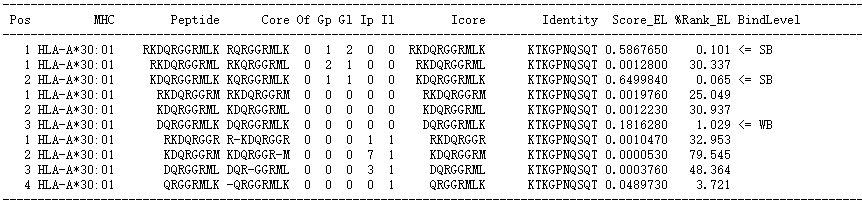

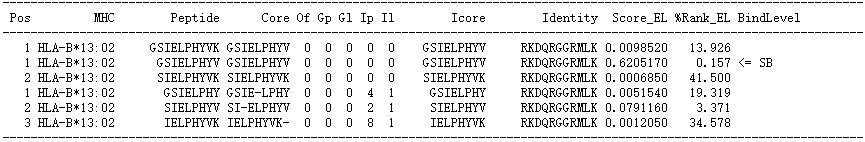
**
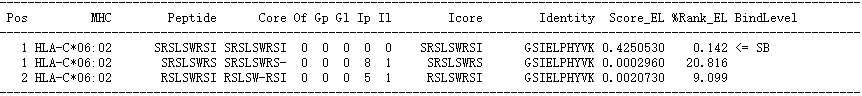
**
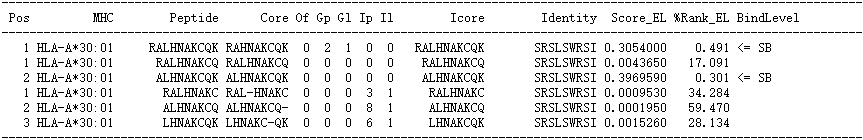

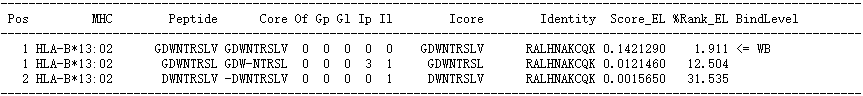

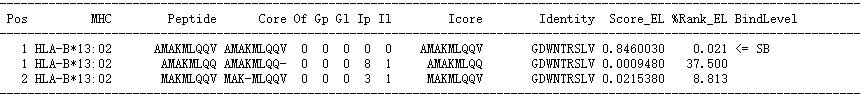

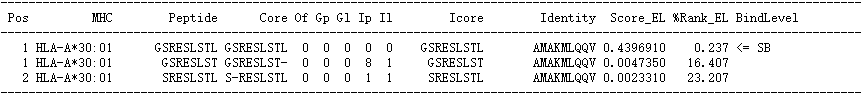
**
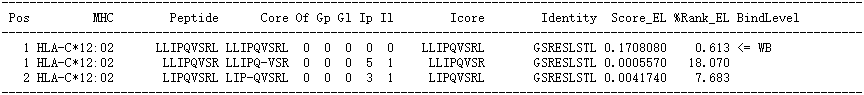
**
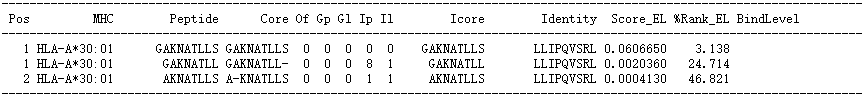

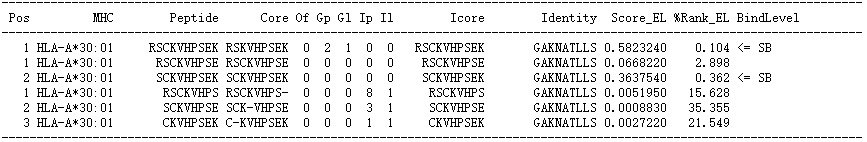

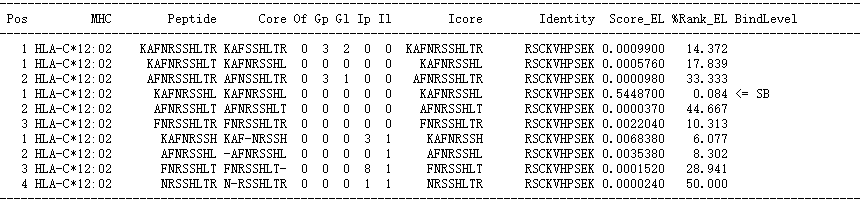

Supplement: Supplementary file 2 [file Table2.docx]
